# Supplementary figures and images for: Tumor-secreted clusterin promotes cachectic fat wasting via disrupting circadian gene expression and adipogenesis
Source: EMBO J. 2025 Dec 17;45(3):856–78. doi: 10.1038/s44318-025-00661-4 (PMC12864892; doi:10.1038/s44318-025-00661-4)

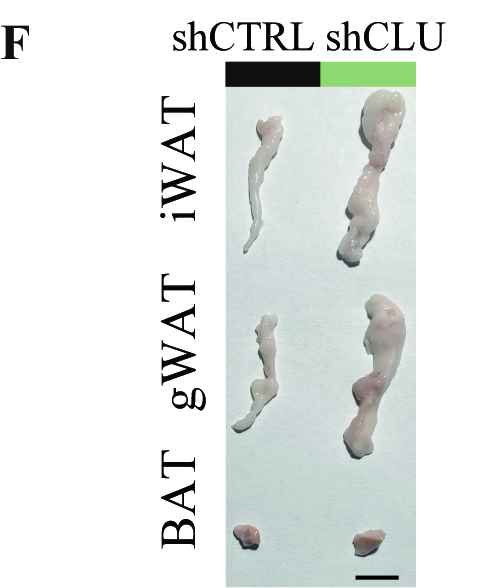

Supplement: Supplementary file 7 — Source data Fig. 1 [file 44318_2025_661_MOESM7_ESM.zip › Figure 1/Figure 1F/Figure 1F.tif]

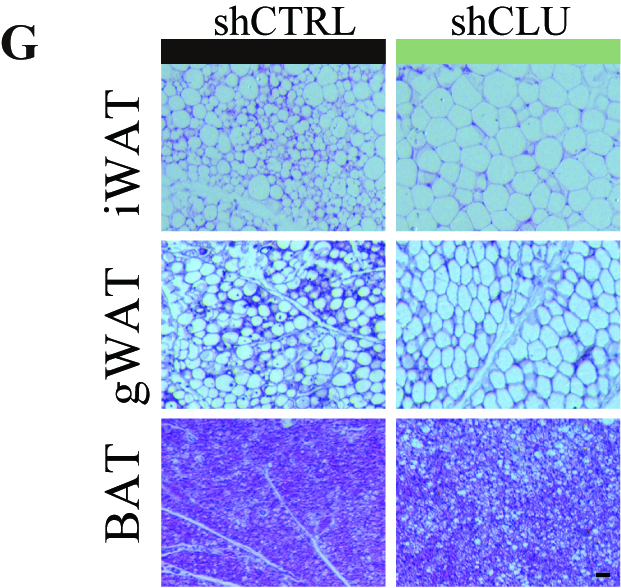

Supplement: Supplementary file 7 — Source data Fig. 1 [file 44318_2025_661_MOESM7_ESM.zip › Figure 1/Figure 1G/Figure 1G.tif]

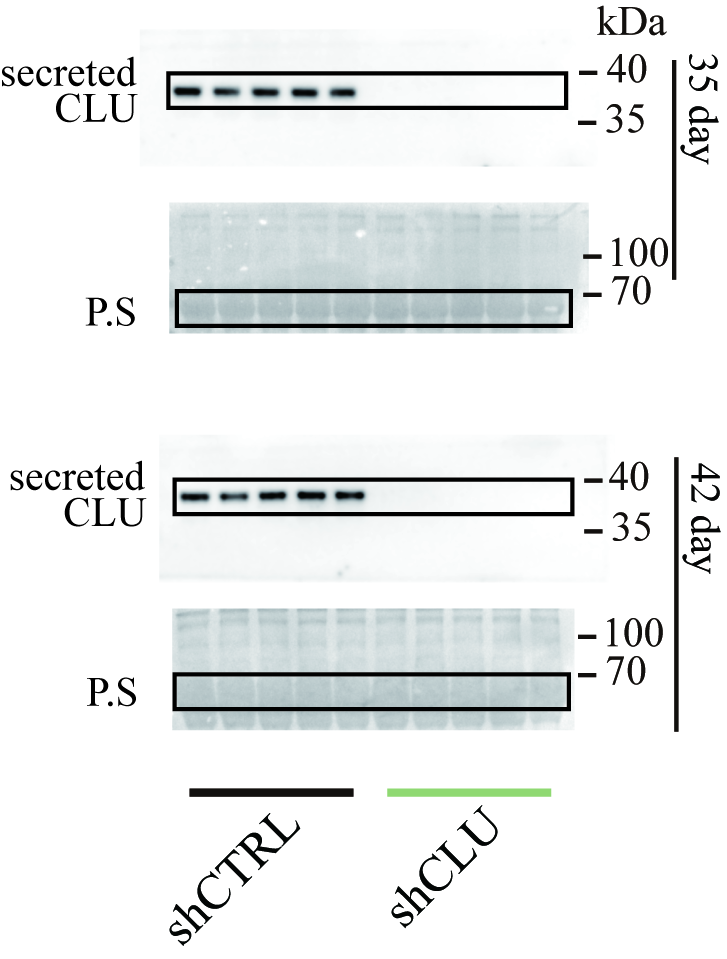

Supplement: Supplementary file 7 — Source data Fig. 1 [file 44318_2025_661_MOESM7_ESM.zip › Figure 1/Figure 1C/Figure 1C.tif]

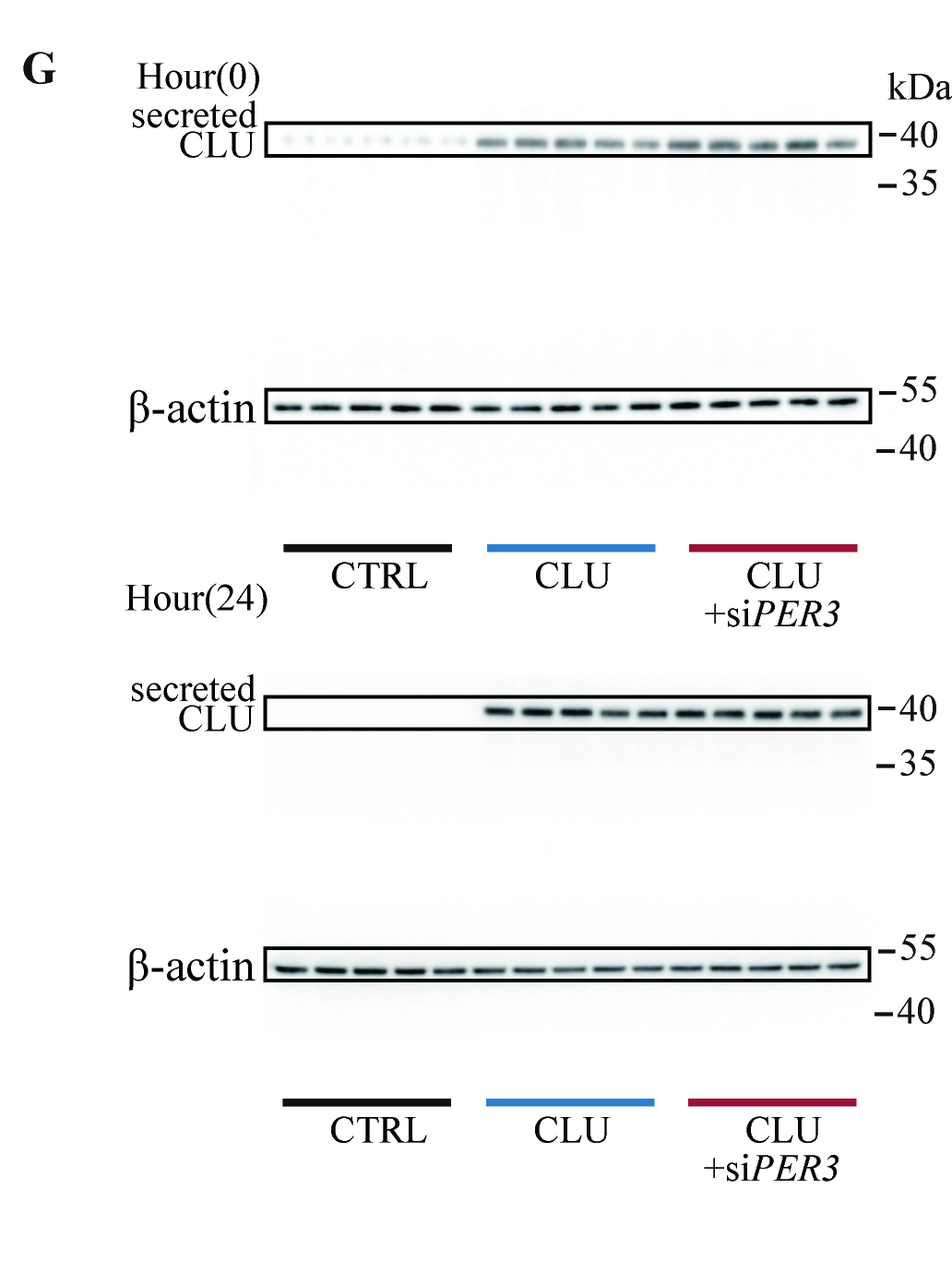

Supplement: Supplementary file 8 — Source data Fig. 2 [file 44318_2025_661_MOESM8_ESM.zip › Figure 2/Figure 2G/Figure 2G.tif]

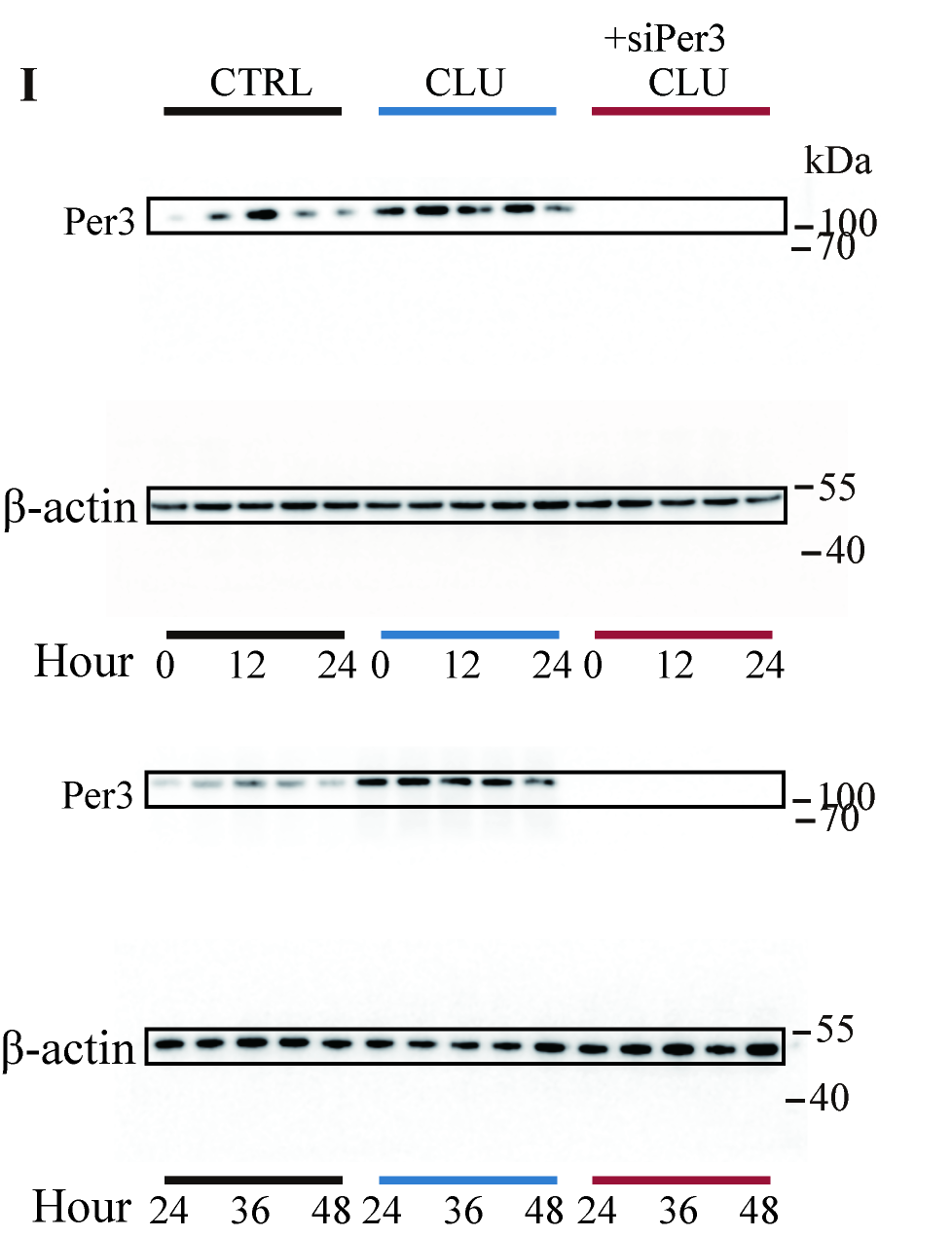

Supplement: Supplementary file 8 — Source data Fig. 2 [file 44318_2025_661_MOESM8_ESM.zip › Figure 2/Figure 2I/Figure 2I.tif]

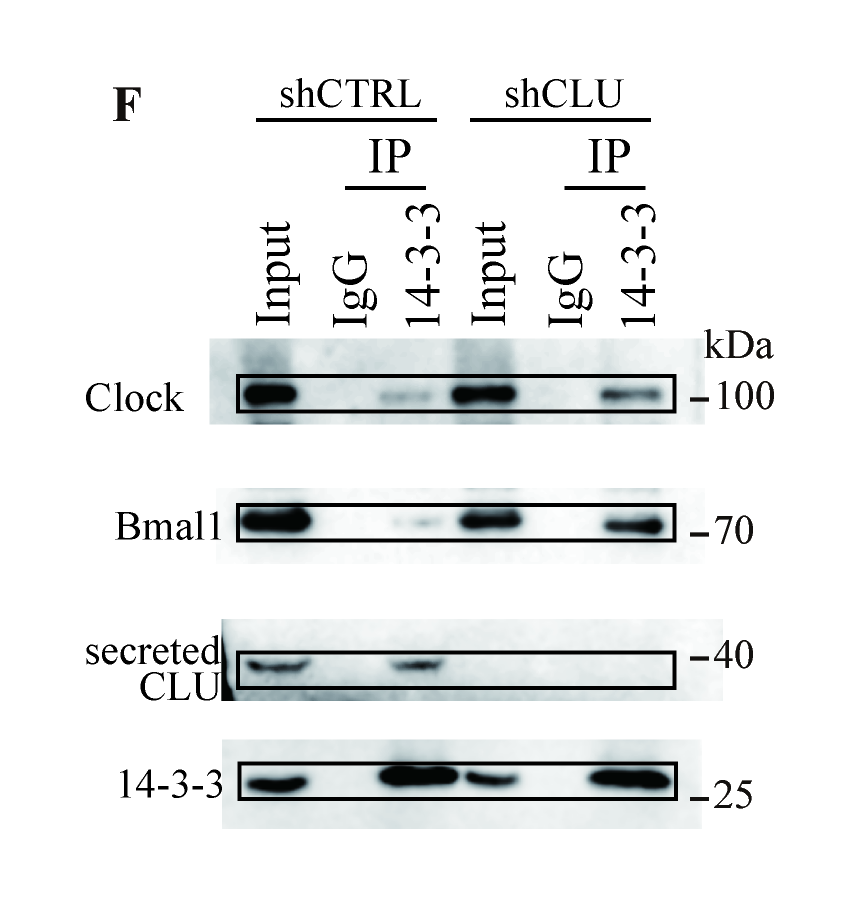

Supplement: Supplementary file 8 — Source data Fig. 2 [file 44318_2025_661_MOESM8_ESM.zip › Figure 2/Figure 2F/Figure 2F.tif]

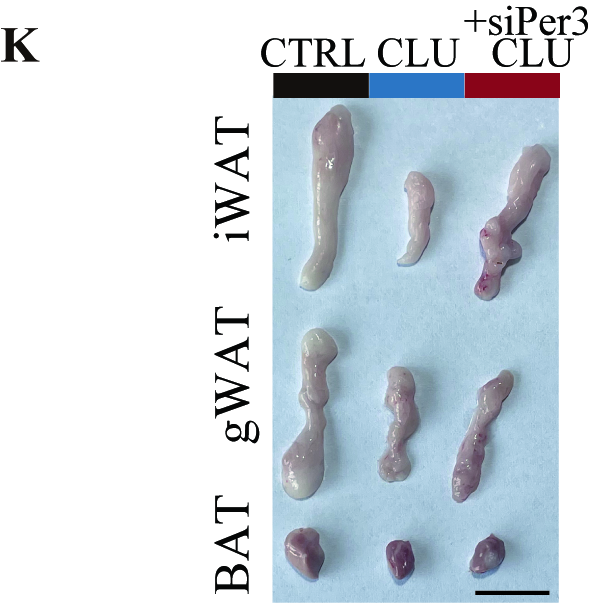

Supplement: Supplementary file 8 — Source data Fig. 2 [file 44318_2025_661_MOESM8_ESM.zip › Figure 2/Figure 2K/Figure 2K.tif]

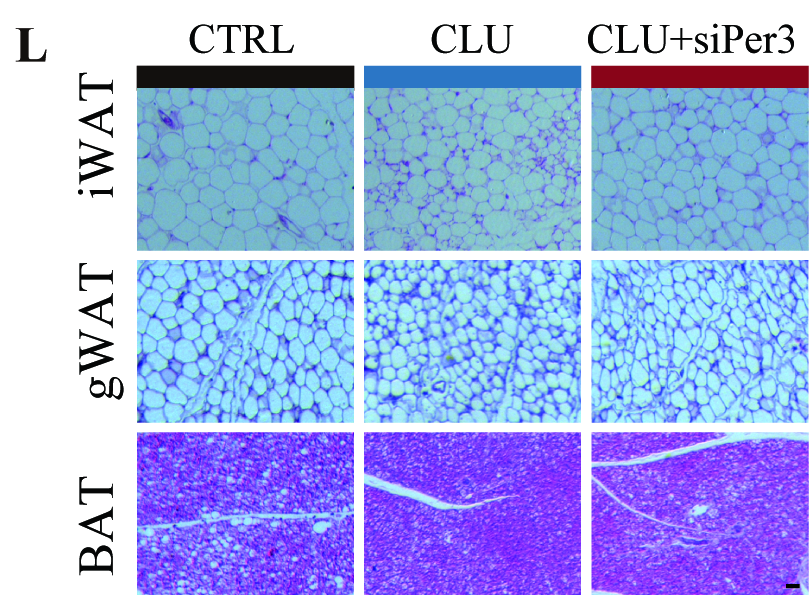

Supplement: Supplementary file 8 — Source data Fig. 2 [file 44318_2025_661_MOESM8_ESM.zip › Figure 2/Figure 2L/Figure 2L.tif]

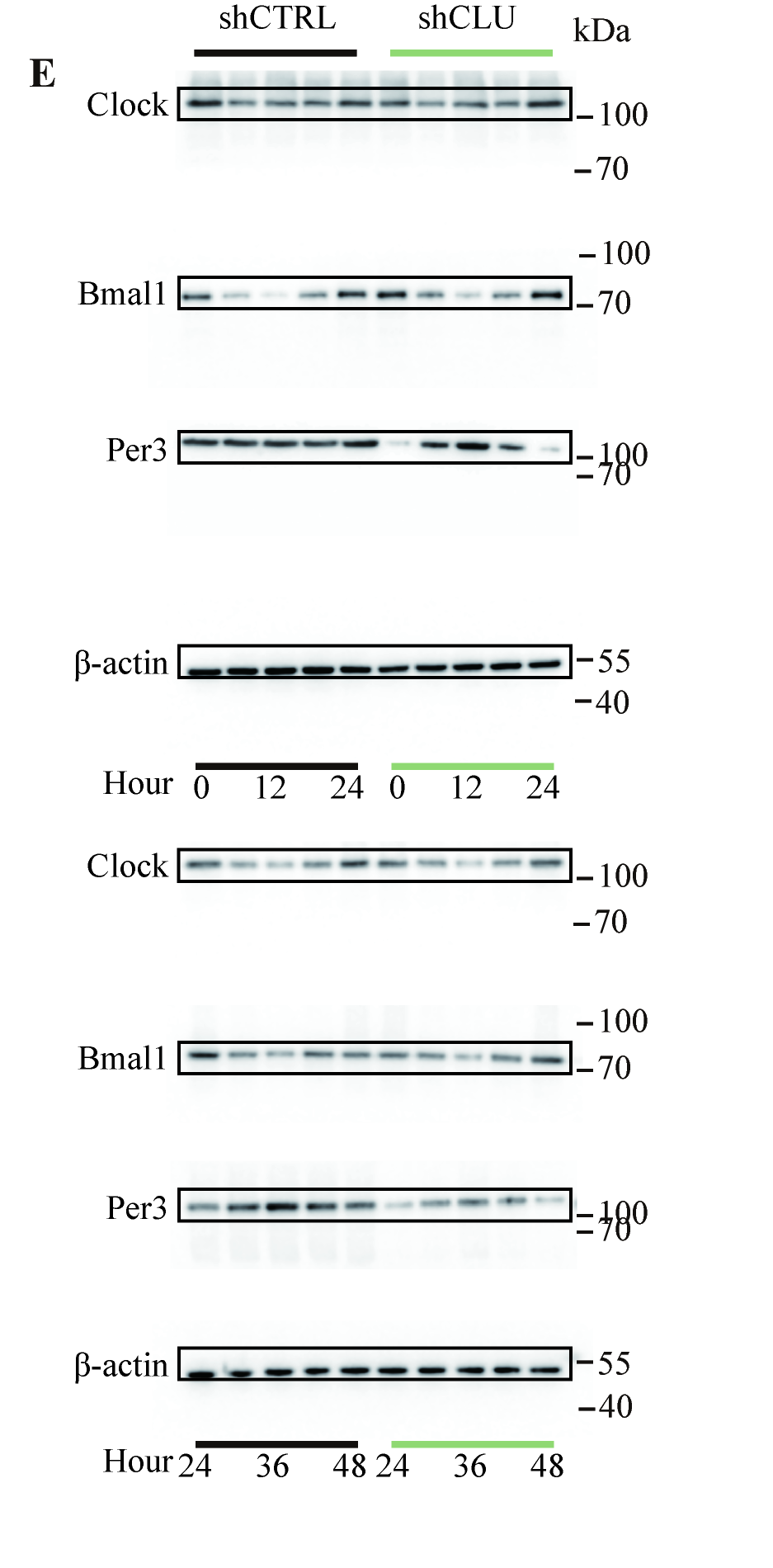

Supplement: Supplementary file 8 — Source data Fig. 2 [file 44318_2025_661_MOESM8_ESM.zip › Figure 2/Figure 2E/Figure 2E.tif]

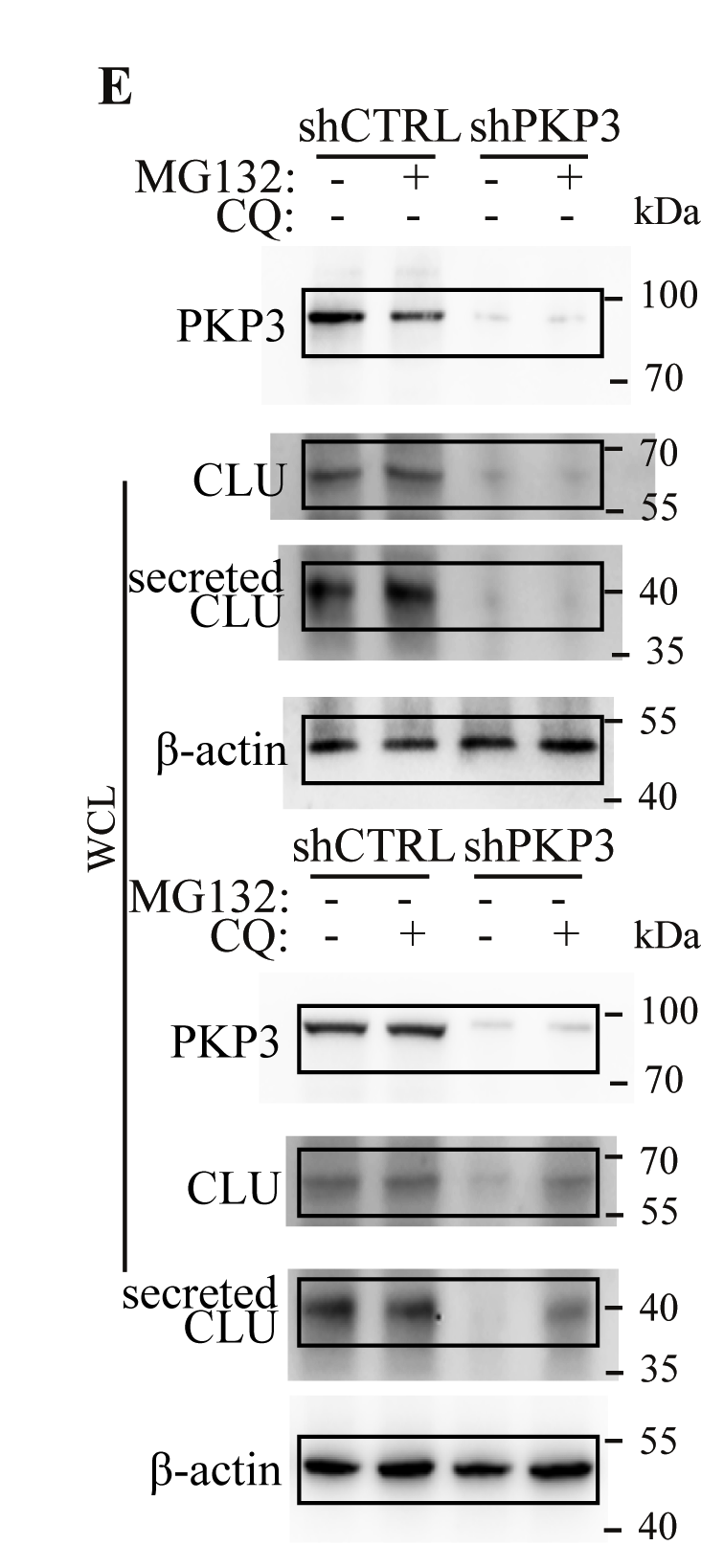

Supplement: Supplementary file 9 — Source data Fig. 3 [file 44318_2025_661_MOESM9_ESM.zip › Figure 3/Figure 3E/Figure 3E.tif]

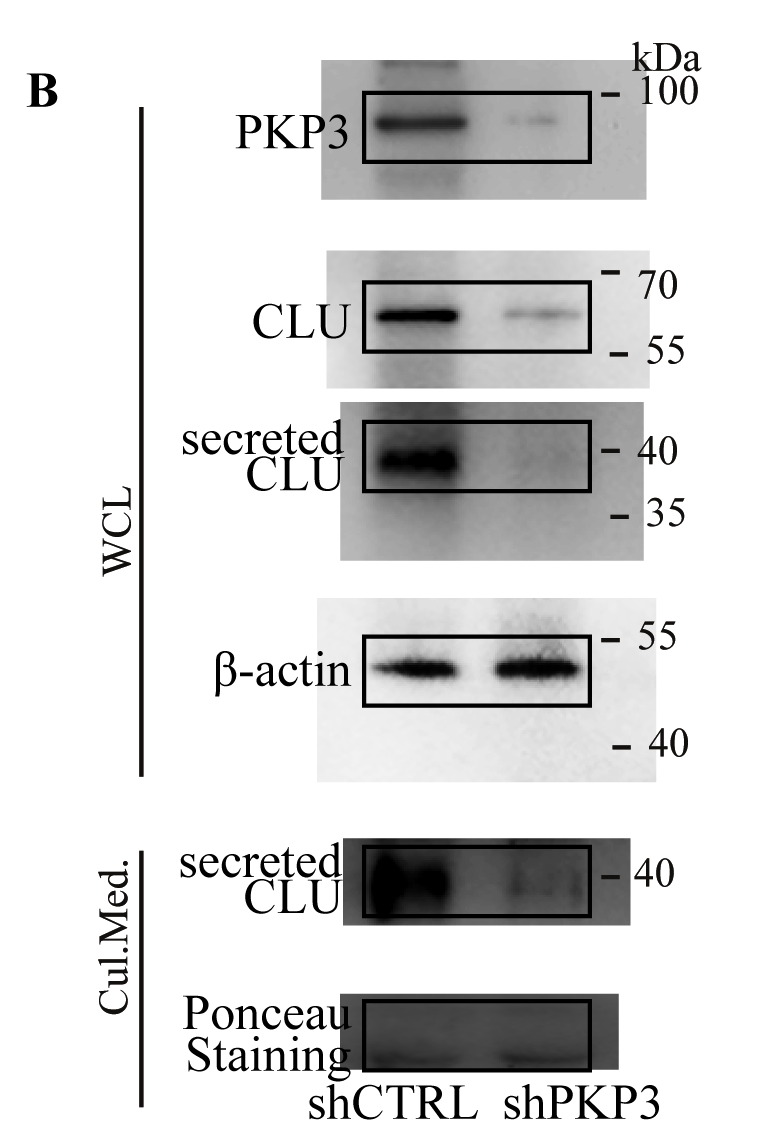

Supplement: Supplementary file 9 — Source data Fig. 3 [file 44318_2025_661_MOESM9_ESM.zip › Figure 3/Figure 3B/Figure 3B.tif]

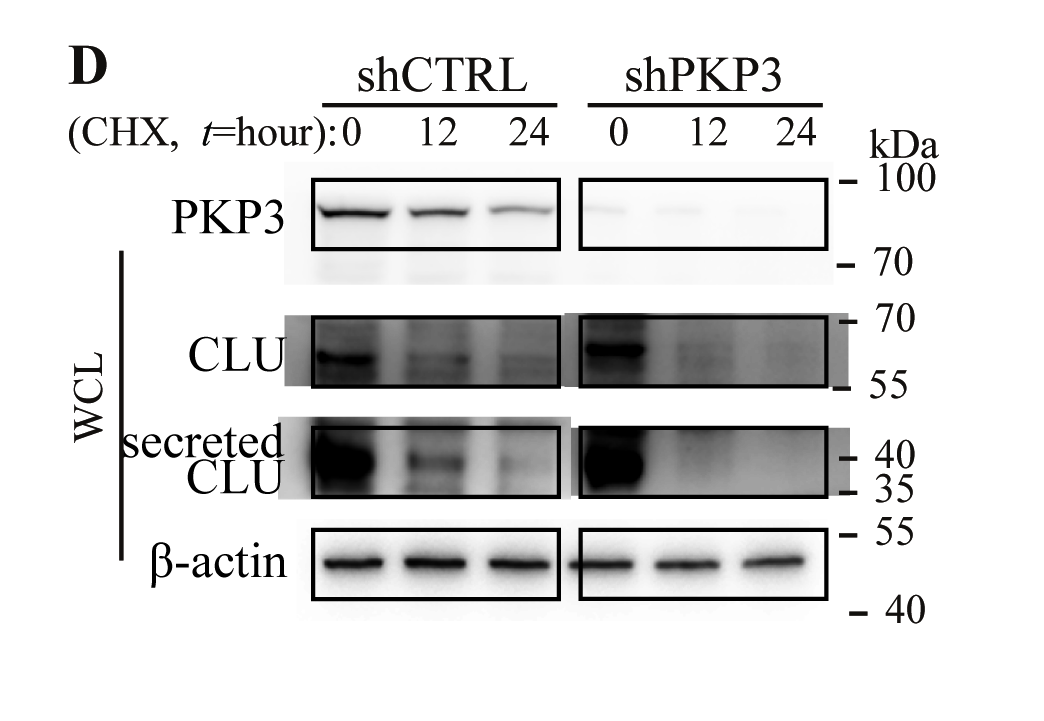

Supplement: Supplementary file 9 — Source data Fig. 3 [file 44318_2025_661_MOESM9_ESM.zip › Figure 3/Figure 3D/Figure 3D.tif]

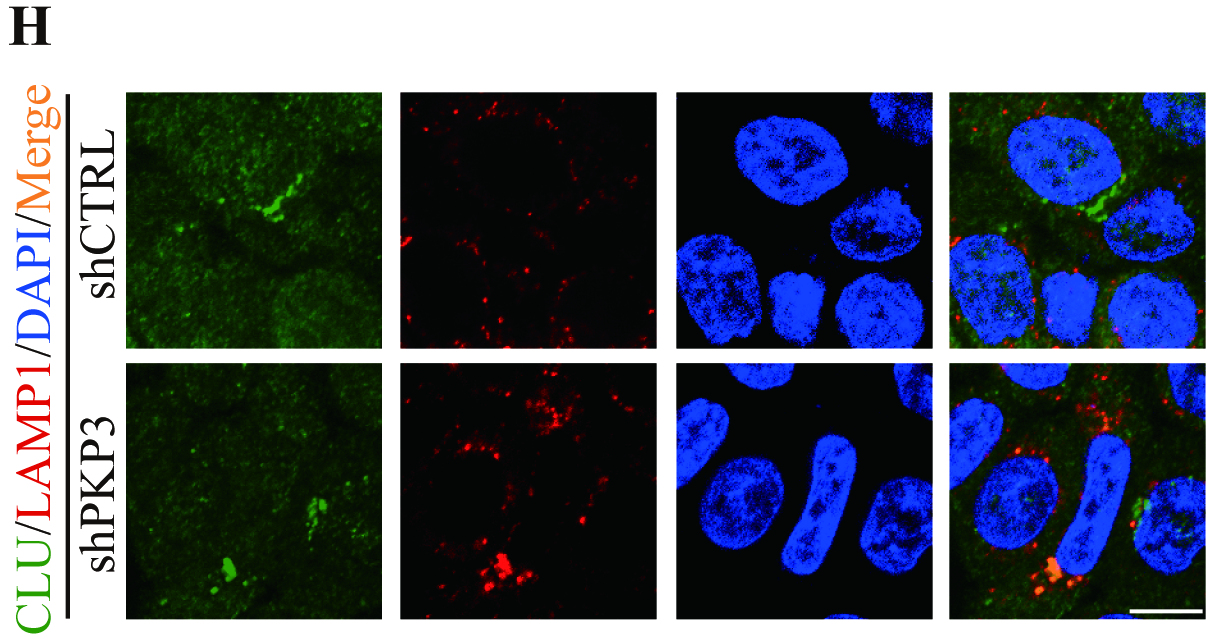

Supplement: Supplementary file 9 — Source data Fig. 3 [file 44318_2025_661_MOESM9_ESM.zip › Figure 3/Figure 3H/Figure 3H.tif]

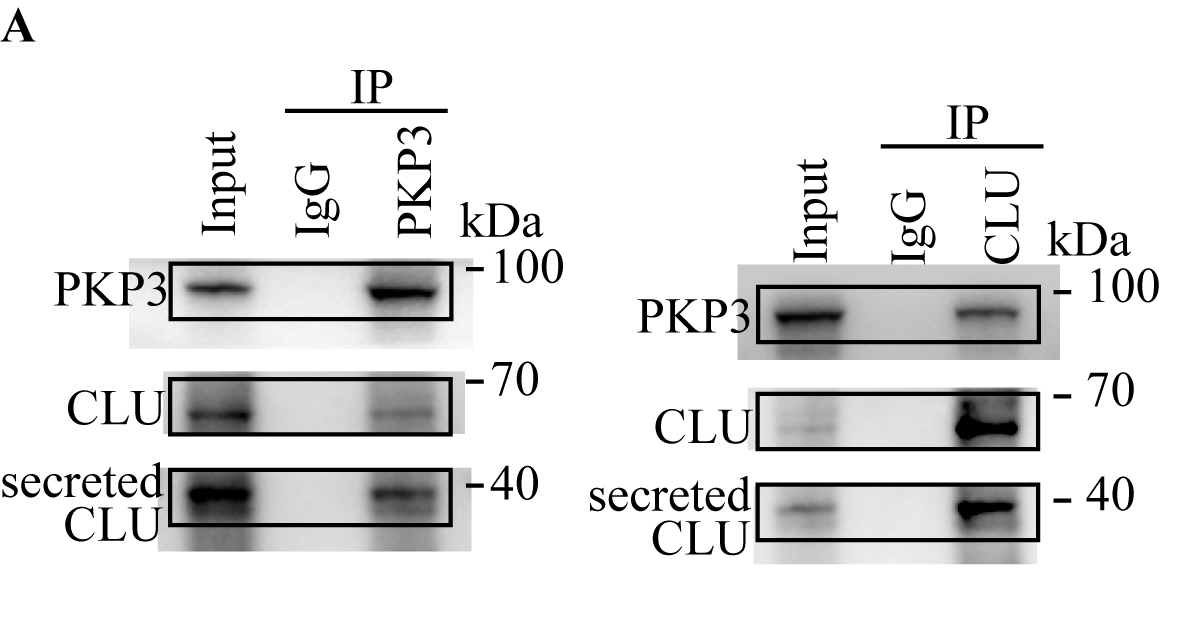

Supplement: Supplementary file 9 — Source data Fig. 3 [file 44318_2025_661_MOESM9_ESM.zip › Figure 3/Figure 3A/Figure 3A.tif]

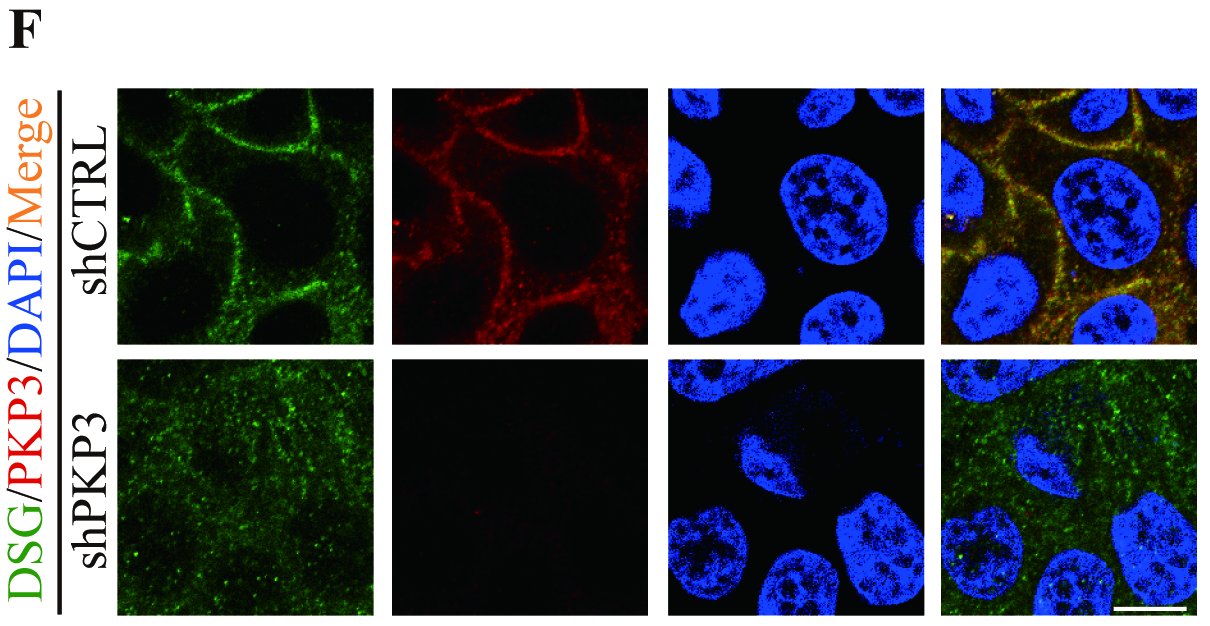

Supplement: Supplementary file 9 — Source data Fig. 3 [file 44318_2025_661_MOESM9_ESM.zip › Figure 3/Figure 3F/Figure 3F.tif]

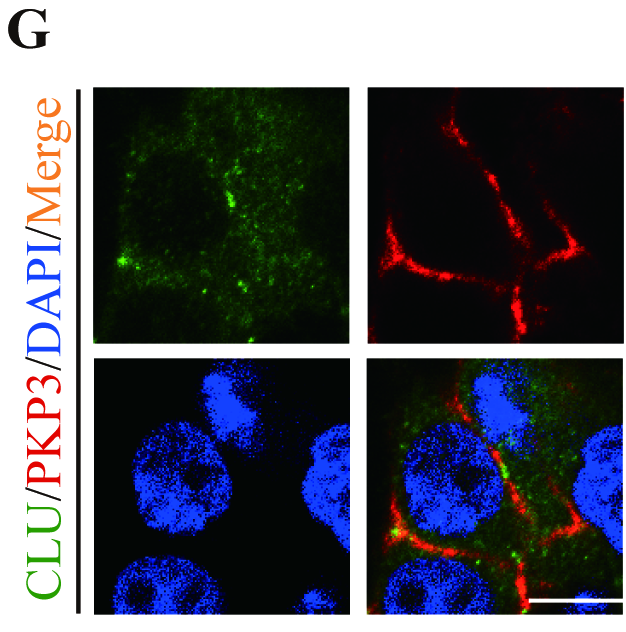

Supplement: Supplementary file 9 — Source data Fig. 3 [file 44318_2025_661_MOESM9_ESM.zip › Figure 3/Figure 3G/Figure 3G.tif]

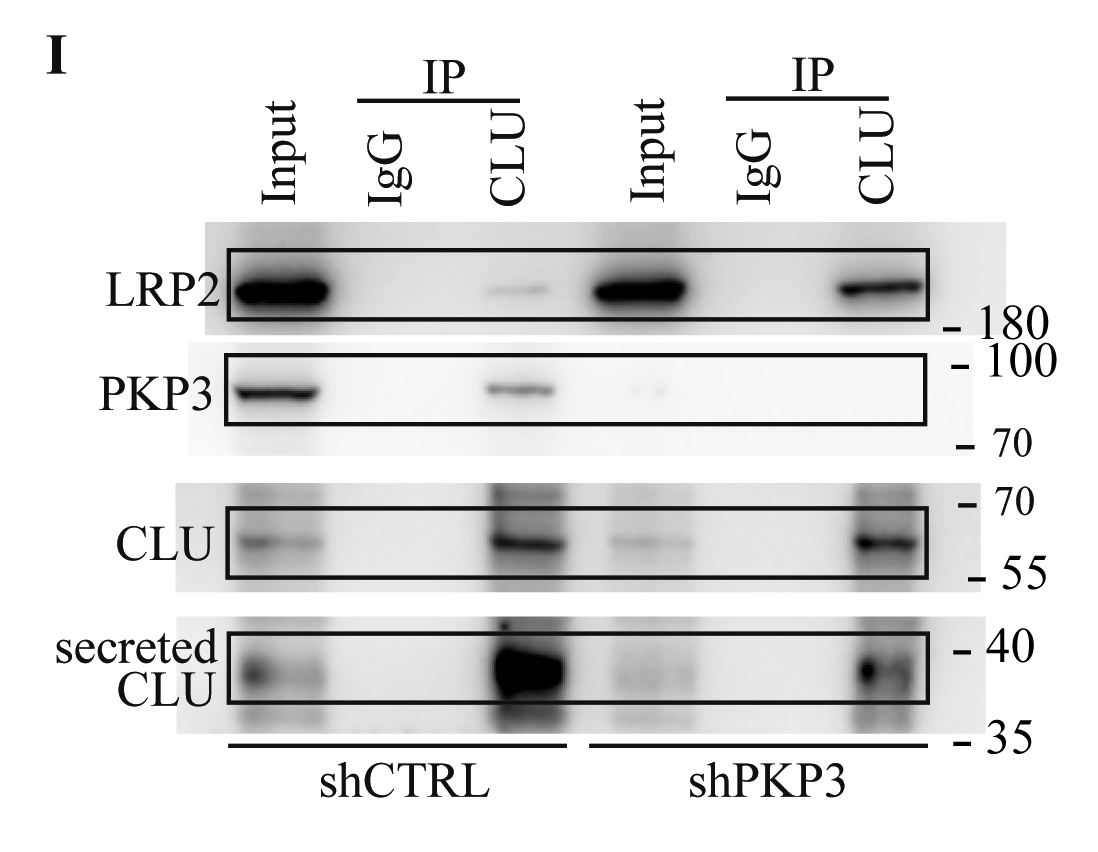

Supplement: Supplementary file 9 — Source data Fig. 3 [file 44318_2025_661_MOESM9_ESM.zip › Figure 3/Figure 3I/Figure 3I.tif]

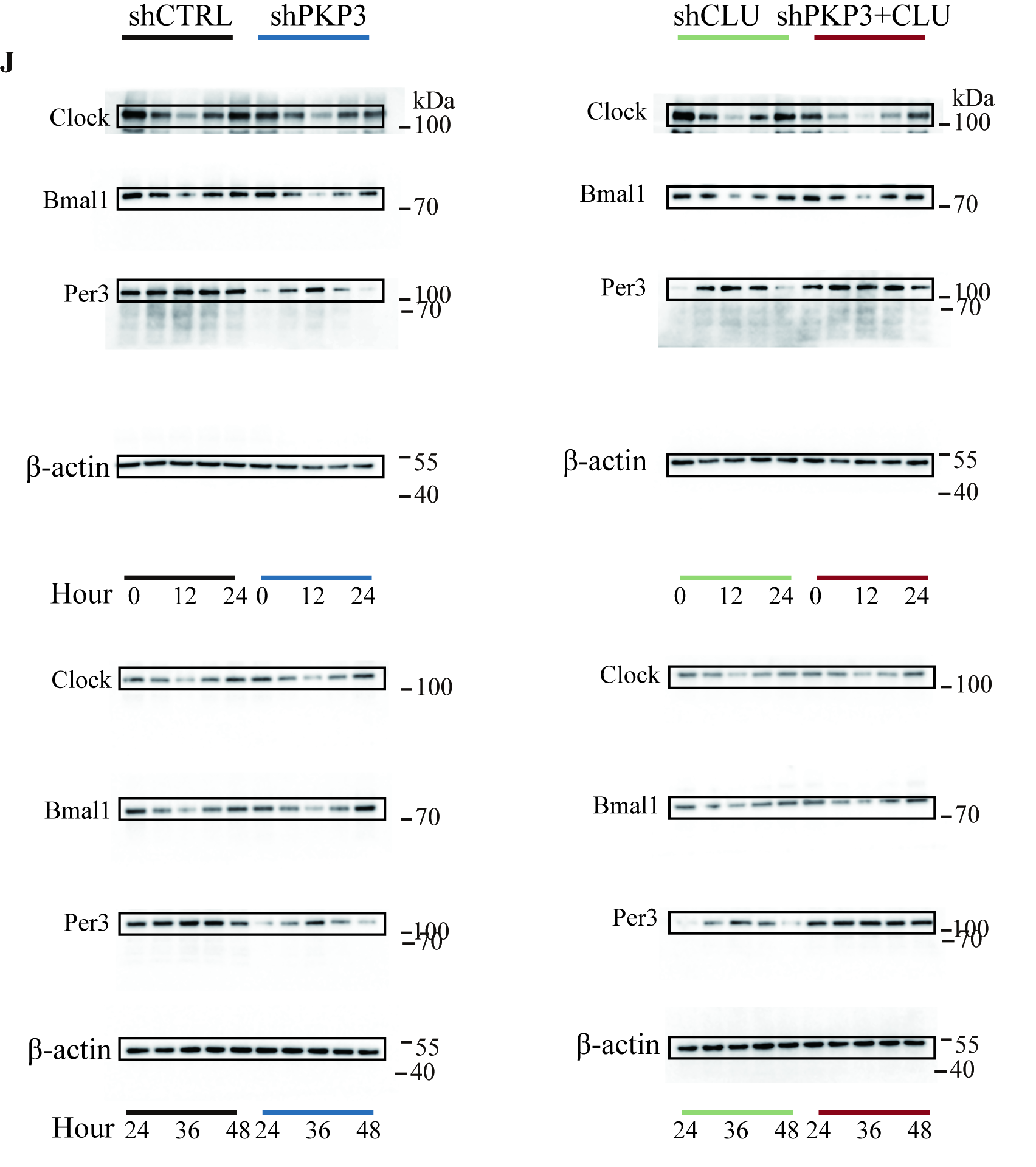

Supplement: Supplementary file 10 — Source data Fig. 4 [file 44318_2025_661_MOESM10_ESM.zip › Figure 4/Figure 4J/Figure 4J.tif]

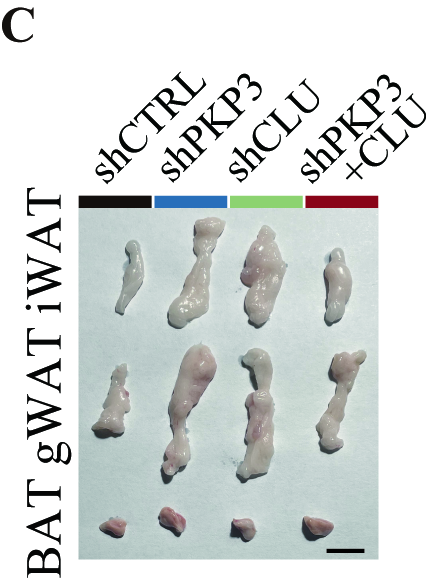

Supplement: Supplementary file 10 — Source data Fig. 4 [file 44318_2025_661_MOESM10_ESM.zip › Figure 4/Figure 4C/Figure 4C.tif]

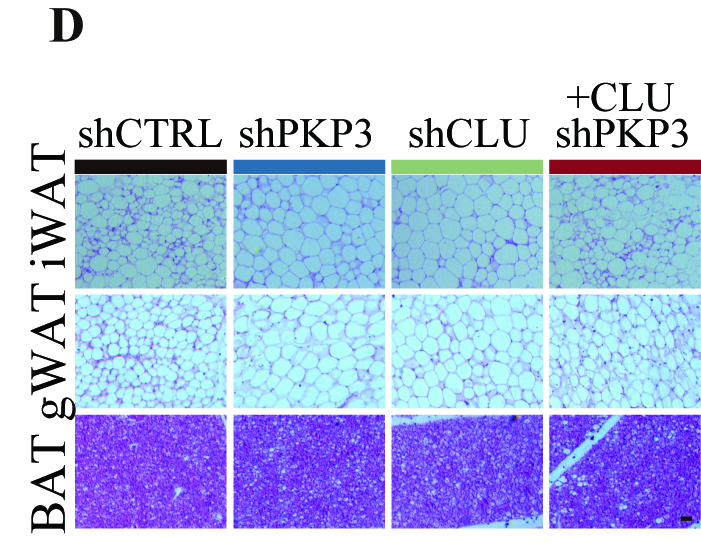

Supplement: Supplementary file 10 — Source data Fig. 4 [file 44318_2025_661_MOESM10_ESM.zip › Figure 4/Figure 4D/Figure 4D.tif]

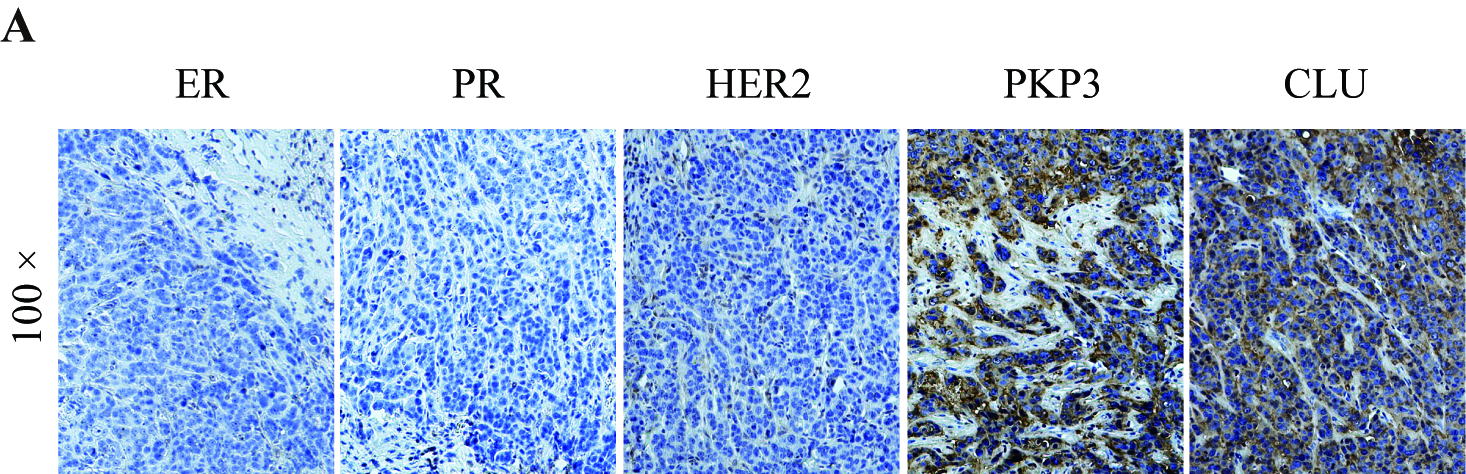

Supplement: Supplementary file 11 — Source data Fig. 5 [file 44318_2025_661_MOESM11_ESM.zip › Figure 5/Figure 5A/Figure 5A.tif]

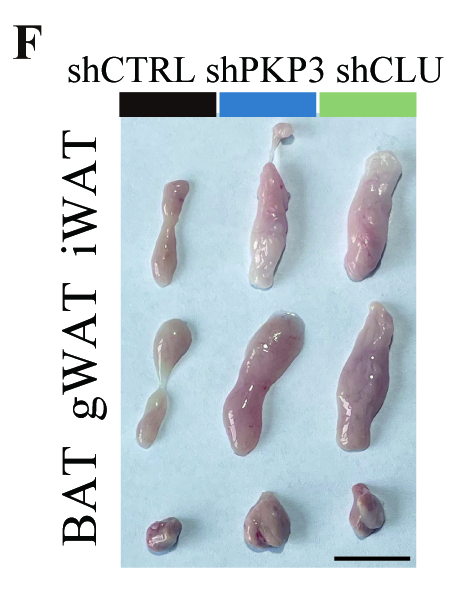

Supplement: Supplementary file 12 — Source data Fig. 6 [file 44318_2025_661_MOESM12_ESM.zip › Figure 6/Figure 6F/Figure 6F.tif]

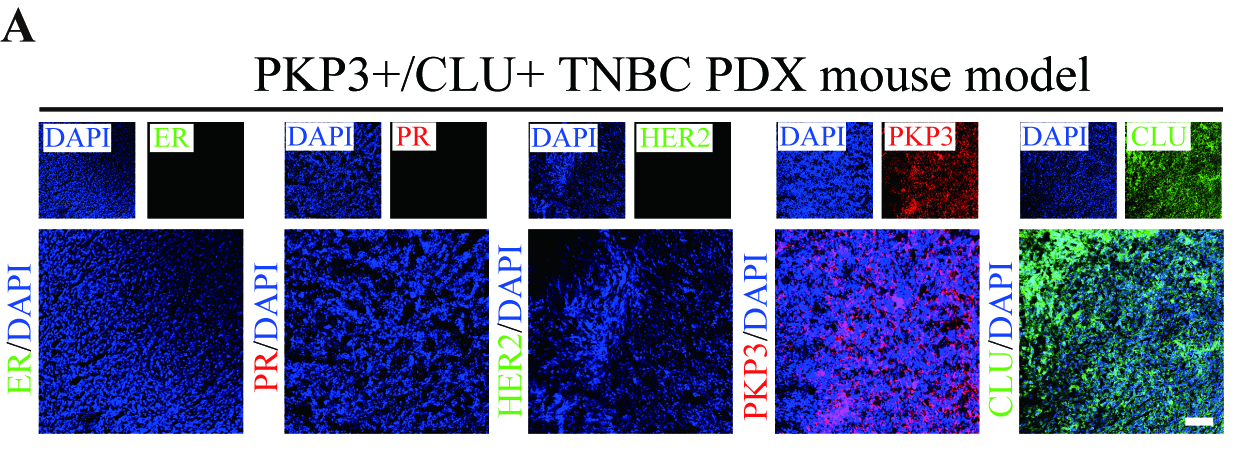

Supplement: Supplementary file 12 — Source data Fig. 6 [file 44318_2025_661_MOESM12_ESM.zip › Figure 6/Figure 6A/Figure 6A.tif]

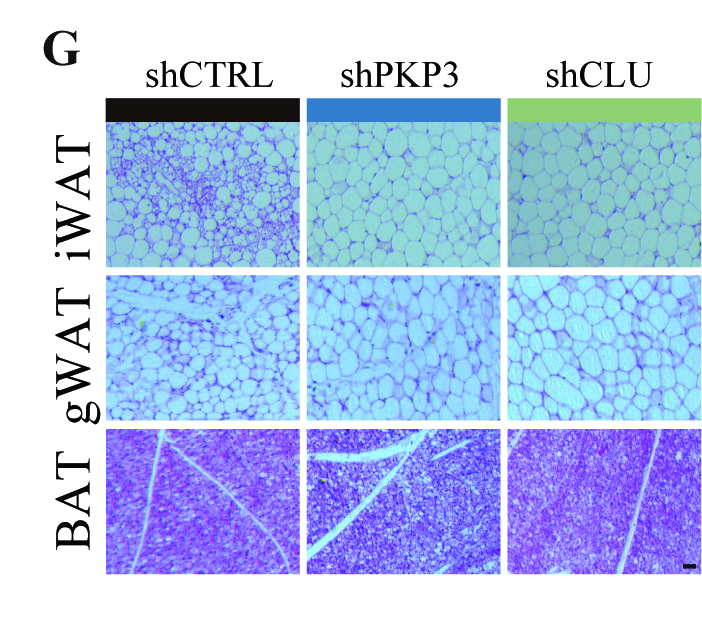

Supplement: Supplementary file 12 — Source data Fig. 6 [file 44318_2025_661_MOESM12_ESM.zip › Figure 6/Figure 6G/Figure 6G.tif]

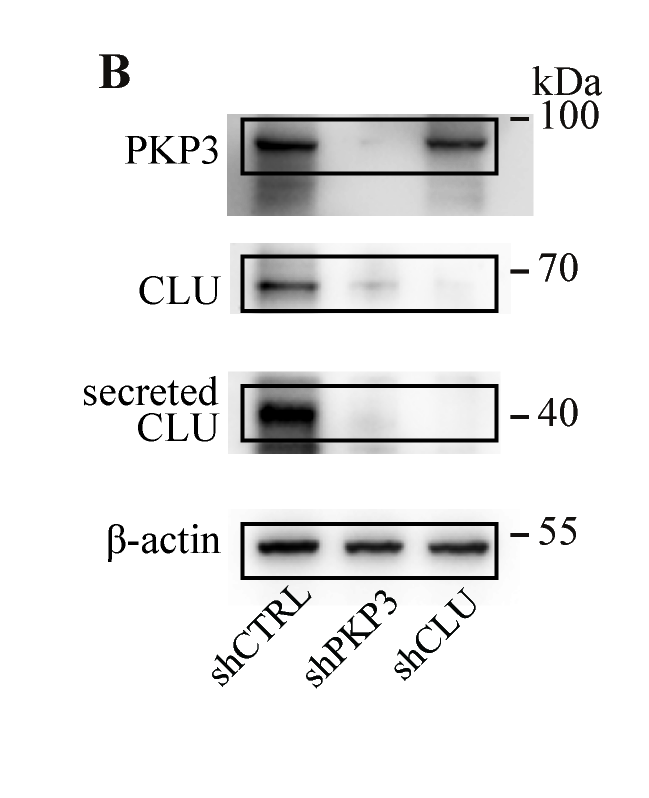

Supplement: Supplementary file 12 — Source data Fig. 6 [file 44318_2025_661_MOESM12_ESM.zip › Figure 6/Figure 6B/Figure 6B.tif]

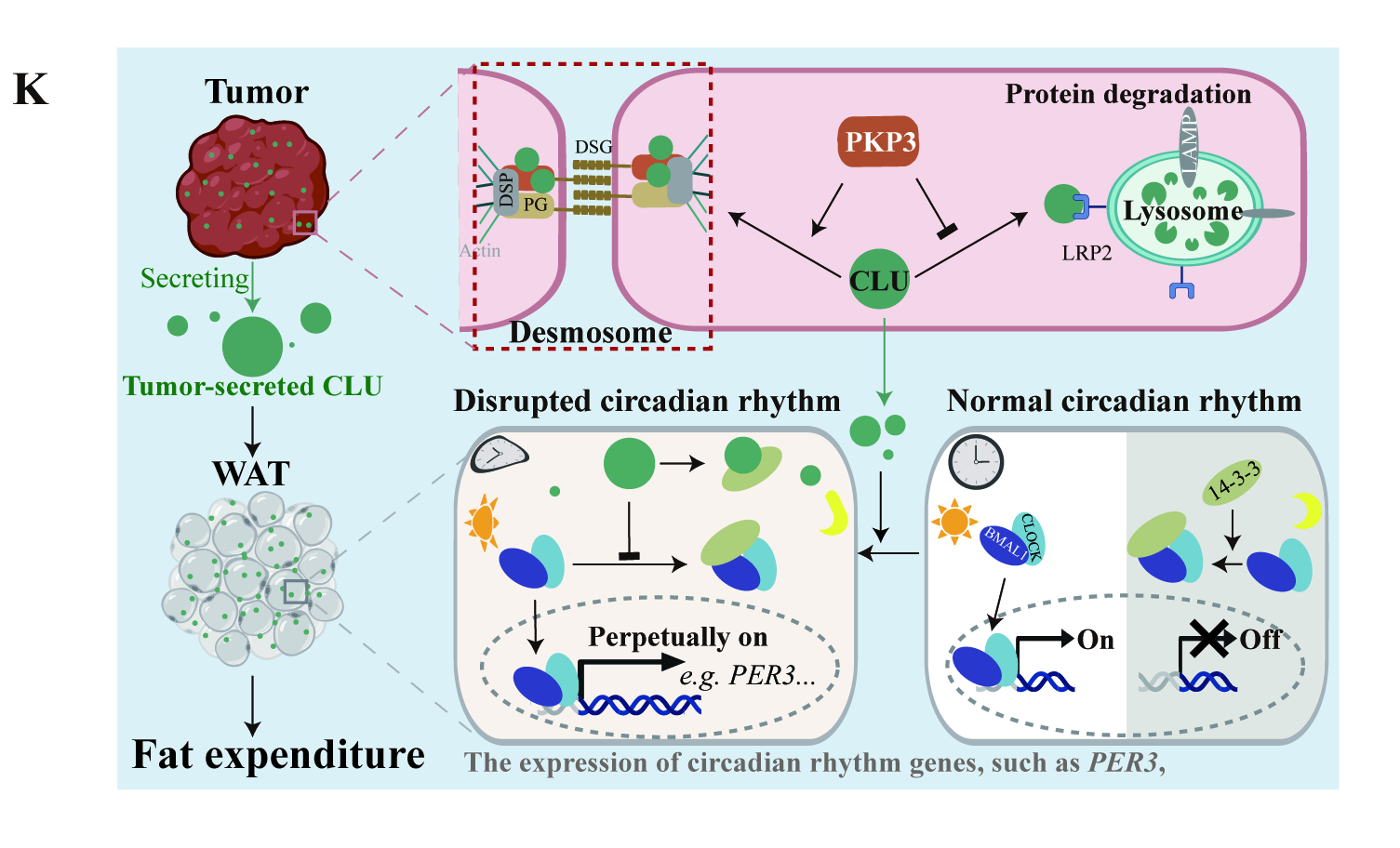

Supplement: Supplementary file 12 — Source data Fig. 6 [file 44318_2025_661_MOESM12_ESM.zip › Figure 6/Figure 6K/Figure 6K.tif]

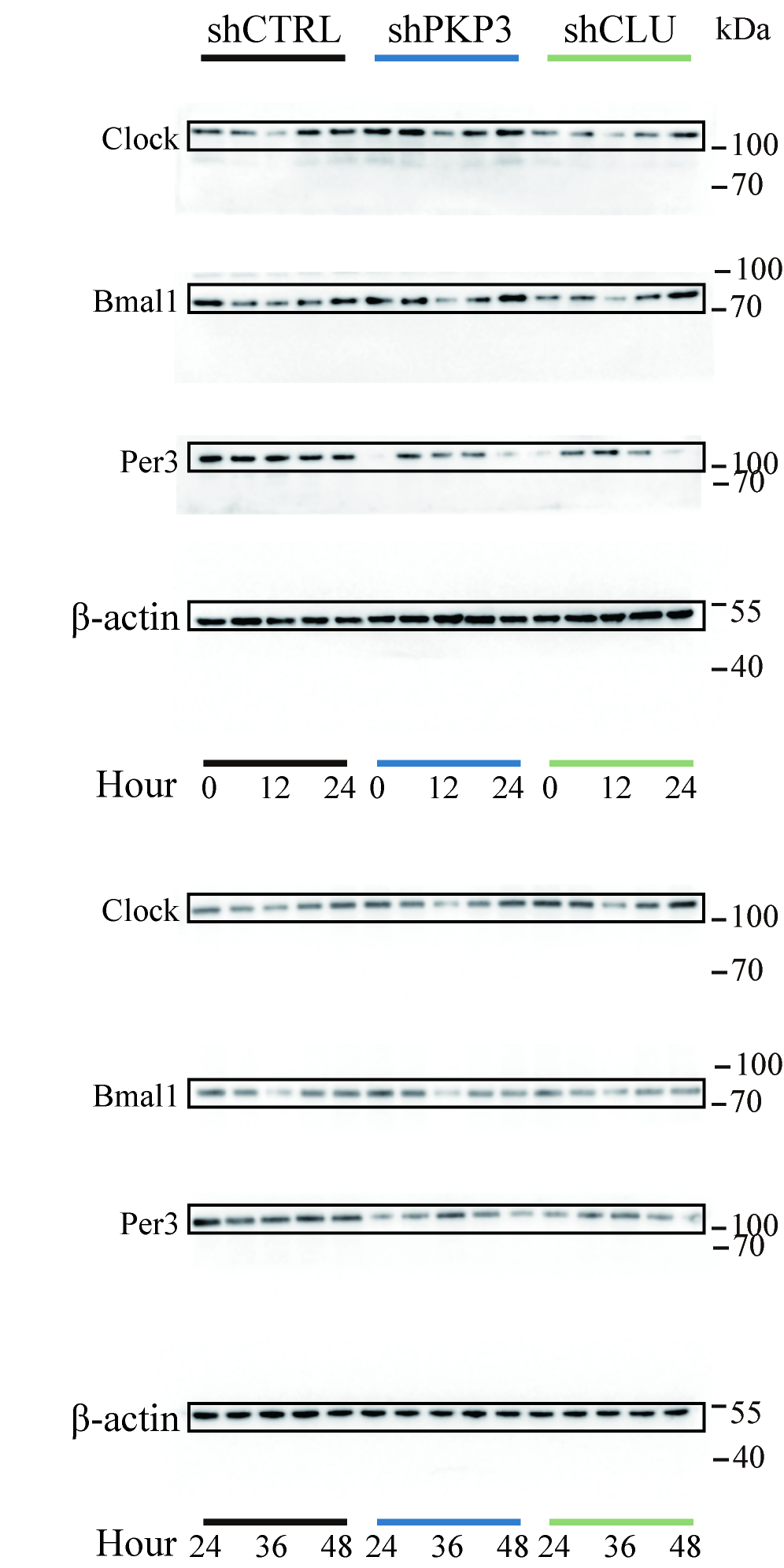

Supplement: Supplementary file 12 — Source data Fig. 6 [file 44318_2025_661_MOESM12_ESM.zip › Figure 6/Figure 6J/Figure 6J.tif]

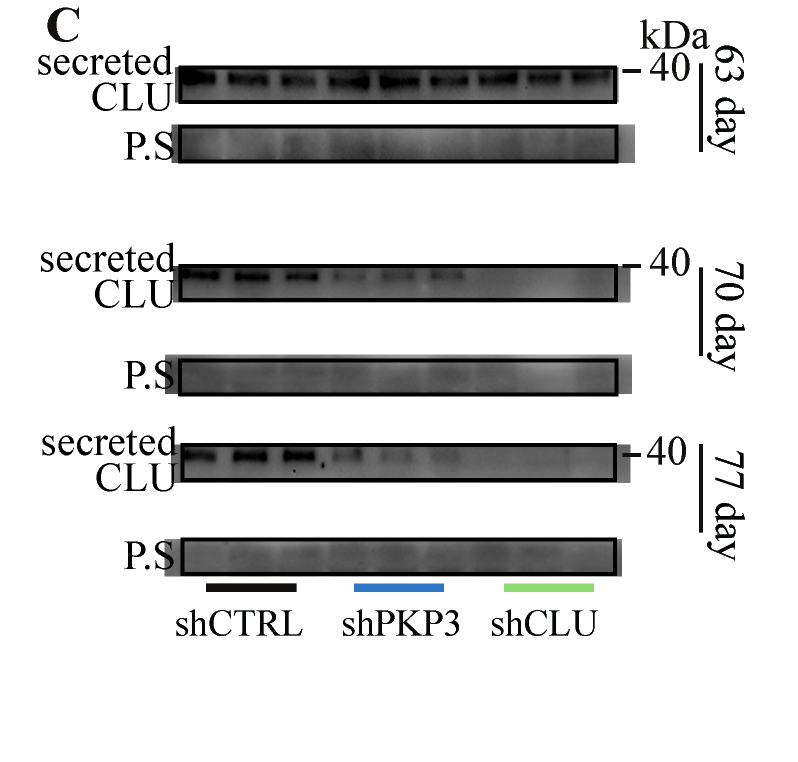

Supplement: Supplementary file 12 — Source data Fig. 6 [file 44318_2025_661_MOESM12_ESM.zip › Figure 6/Figure 6C/Figure 6C.tif]

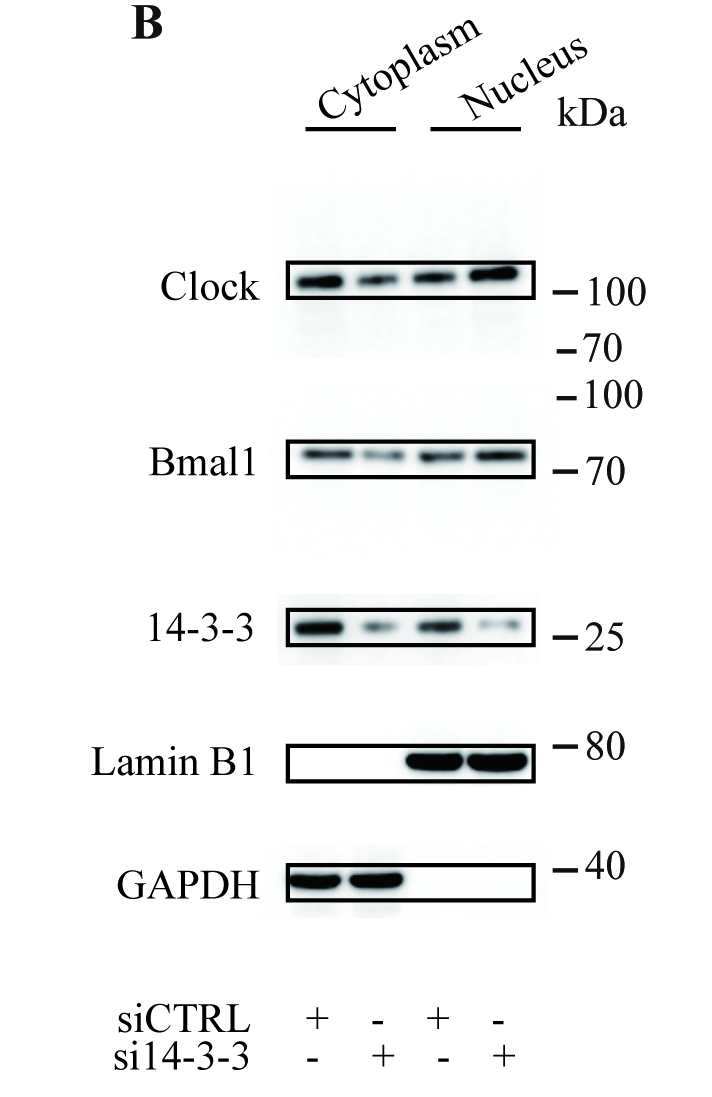

Supplement: Supplementary file 14 — Figure EV2 Source Data [file 44318_2025_661_MOESM14_ESM.zip › Figure EV2/Figure EV2B/Figure EV2B.tif]

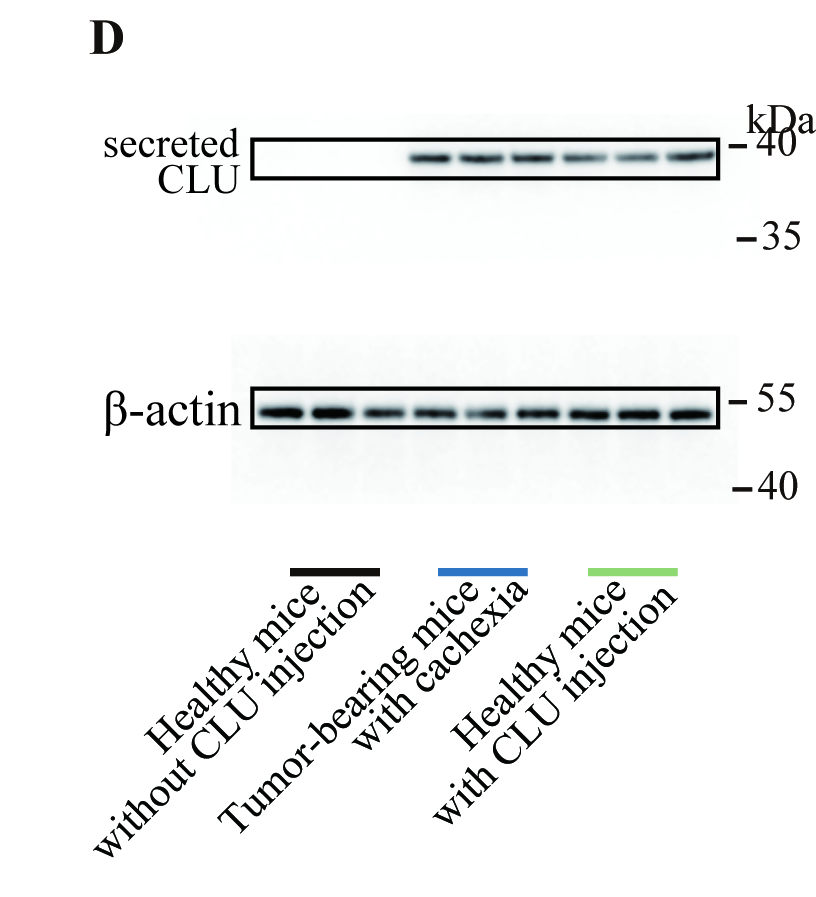

Supplement: Supplementary file 14 — Figure EV2 Source Data [file 44318_2025_661_MOESM14_ESM.zip › Figure EV2/Figure EV2D/Figure EV2D.tif]

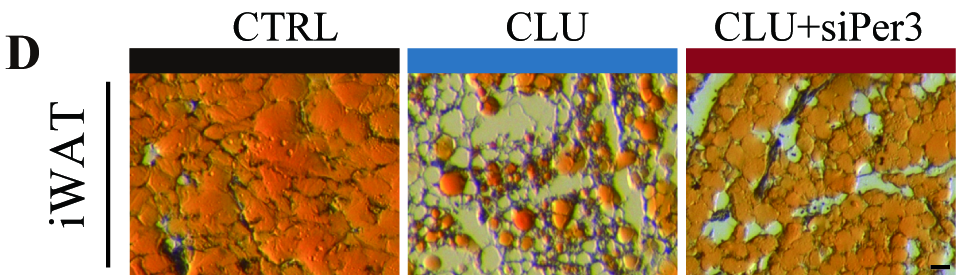

Supplement: Supplementary file 15 — Figure EV3 Source Data [file 44318_2025_661_MOESM15_ESM.zip › Figure EV3/Figure EV3D/Figure EV3D.tif]

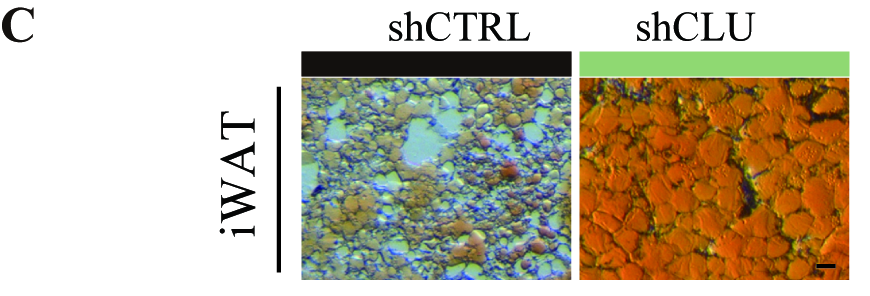

Supplement: Supplementary file 15 — Figure EV3 Source Data [file 44318_2025_661_MOESM15_ESM.zip › Figure EV3/Figure EV3C/Figure EV3C.tif]

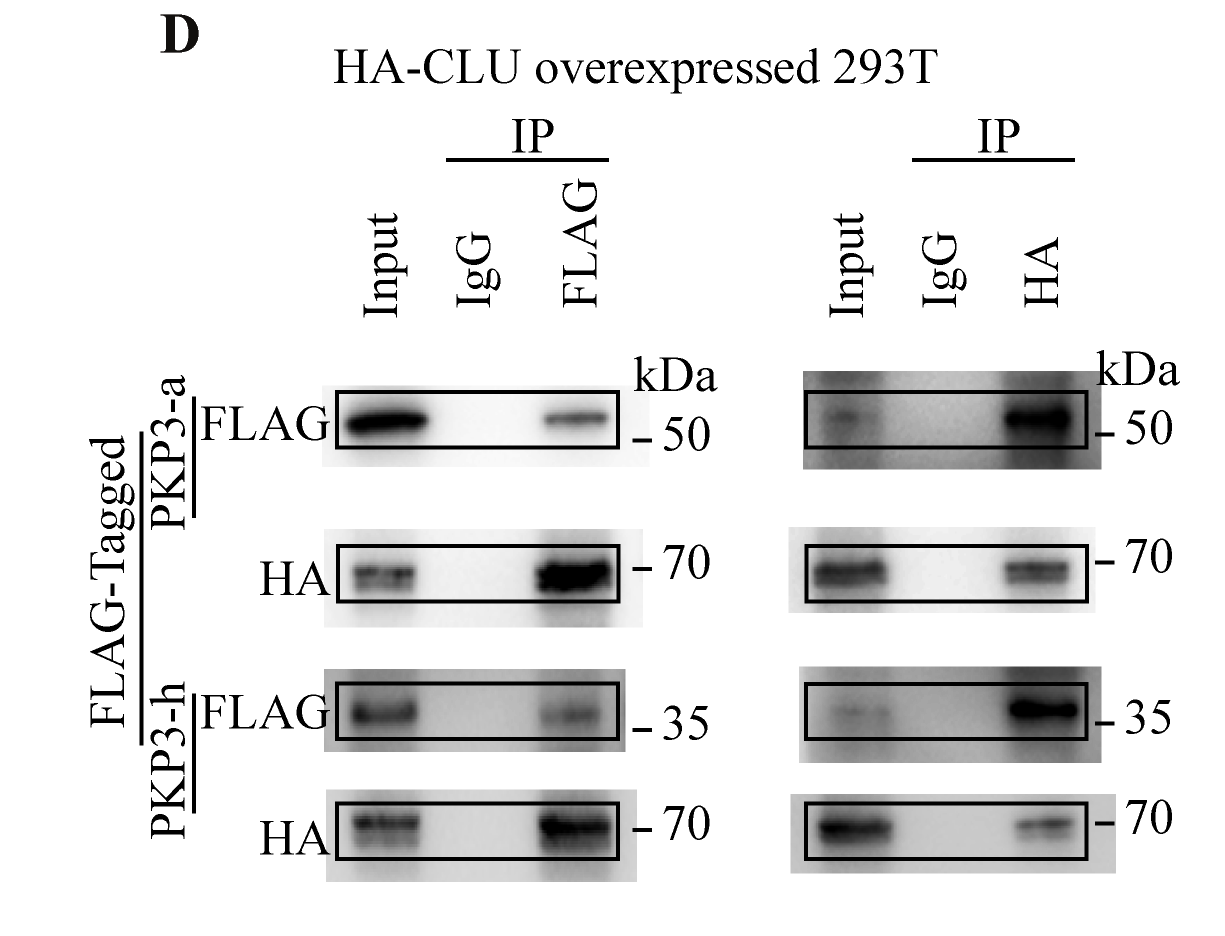

Supplement: Supplementary file 16 — Figure EV4 Source Data [file 44318_2025_661_MOESM16_ESM.zip › Figure EV4/Figure EV4D/Figure EV4D.tif]

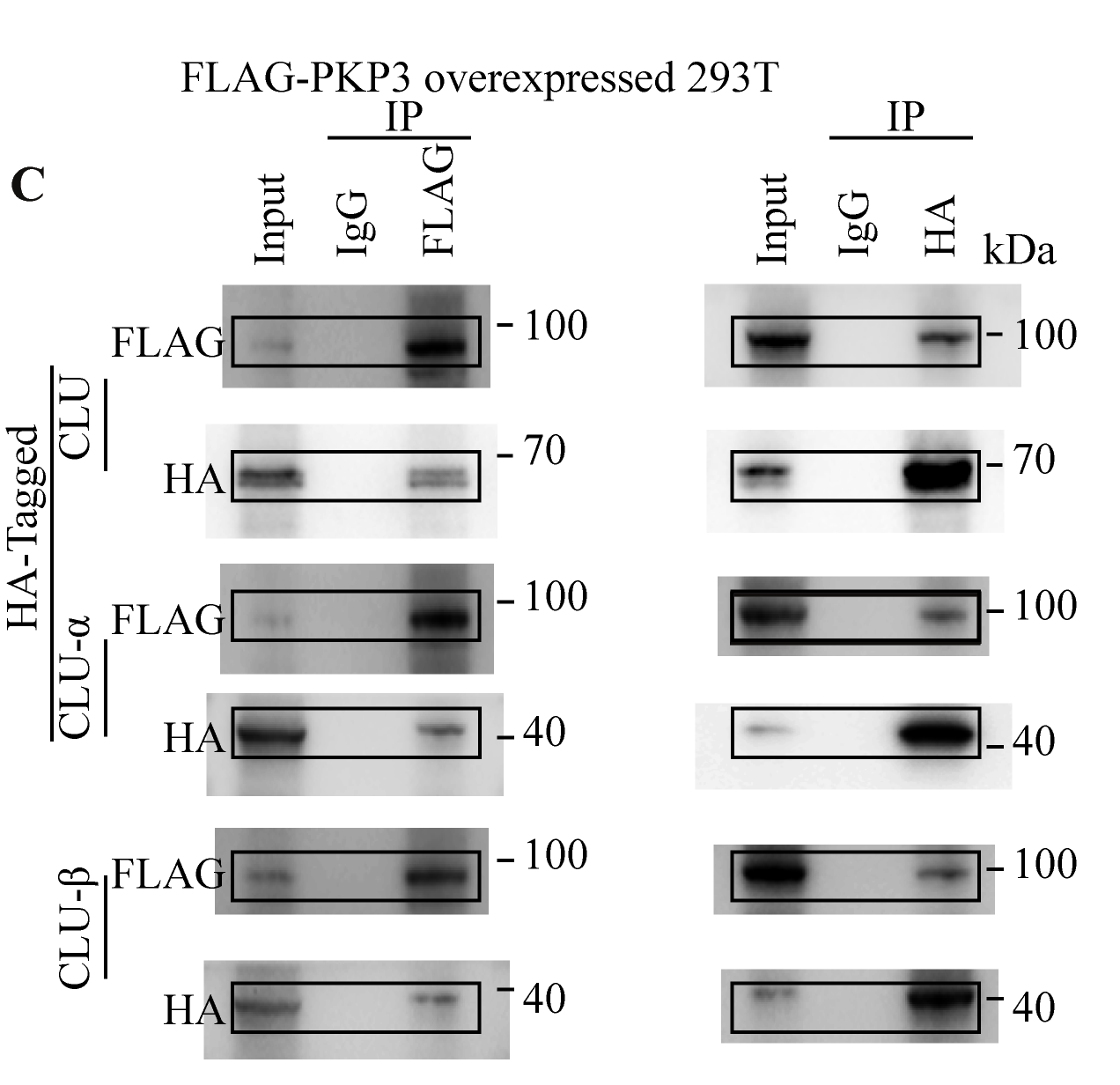

Supplement: Supplementary file 16 — Figure EV4 Source Data [file 44318_2025_661_MOESM16_ESM.zip › Figure EV4/Figure EV4C/Figure EV4C.tif]

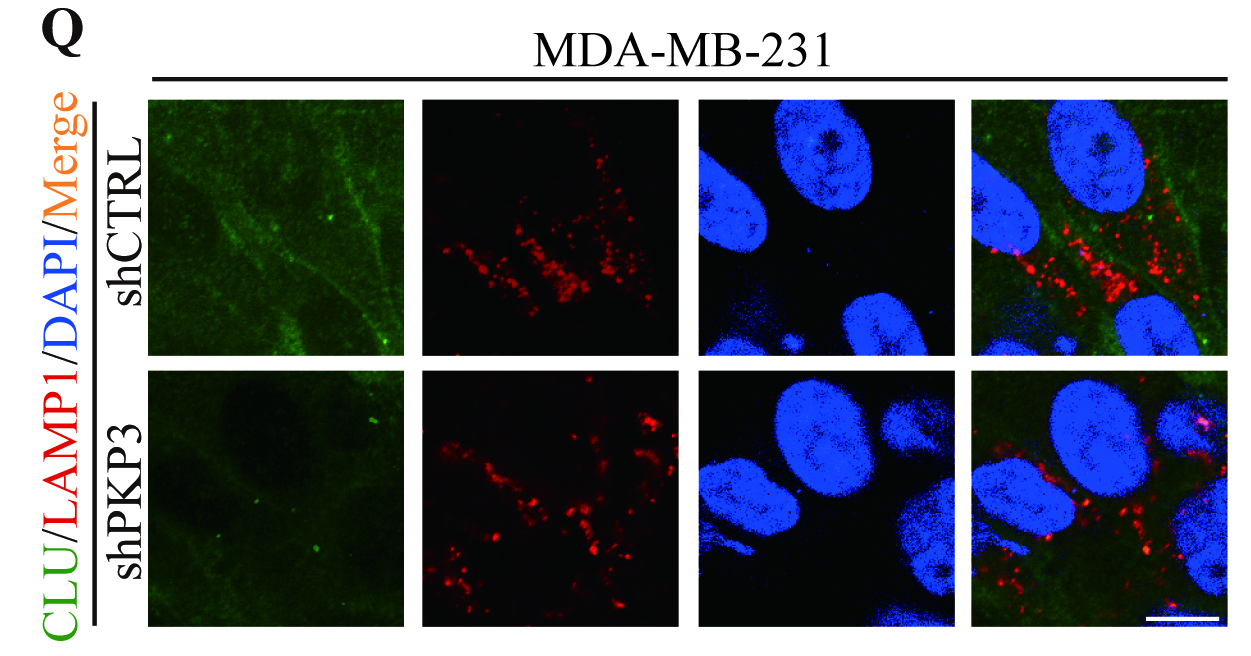

Supplement: Supplementary file 16 — Figure EV4 Source Data [file 44318_2025_661_MOESM16_ESM.zip › Figure EV4/Figure EV4Q/Figure EV4Q.tif]

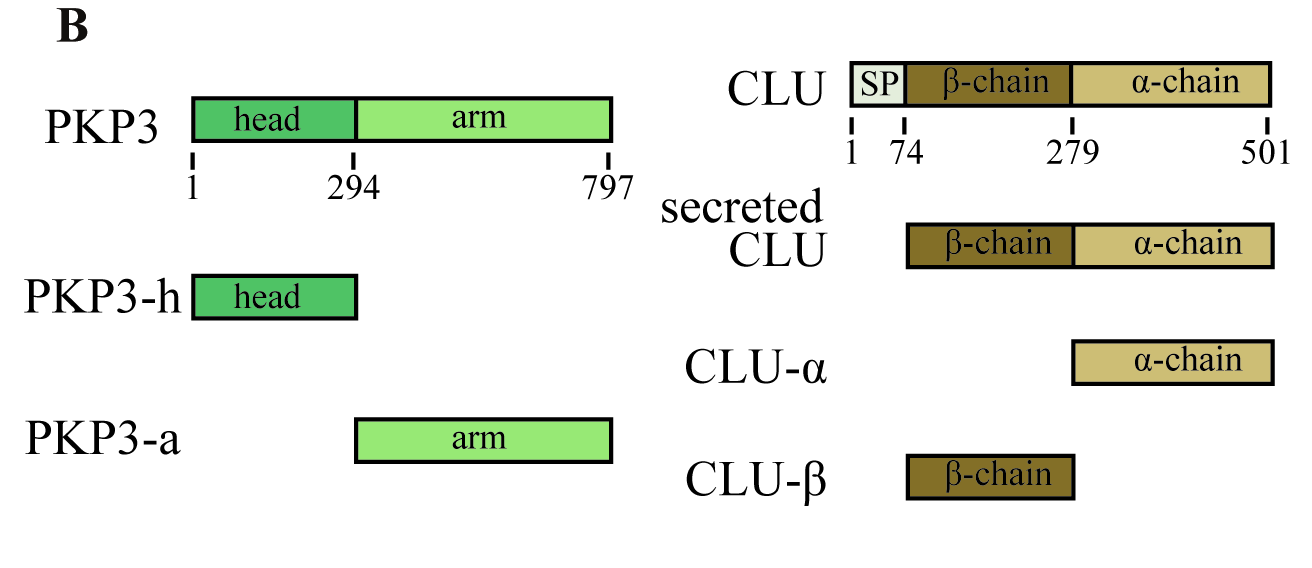

Supplement: Supplementary file 16 — Figure EV4 Source Data [file 44318_2025_661_MOESM16_ESM.zip › Figure EV4/Figure EV4B/Figure EV4B.tif]

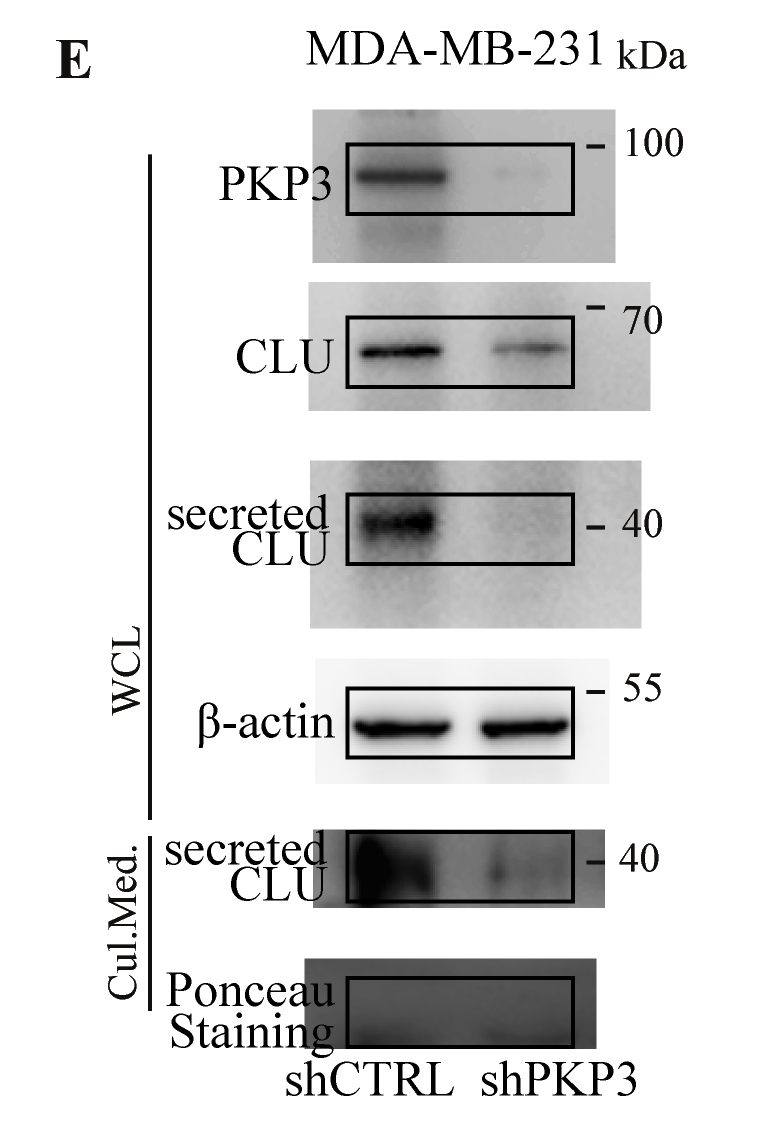

Supplement: Supplementary file 16 — Figure EV4 Source Data [file 44318_2025_661_MOESM16_ESM.zip › Figure EV4/Figure EV4E/Figure EV4E.tif]

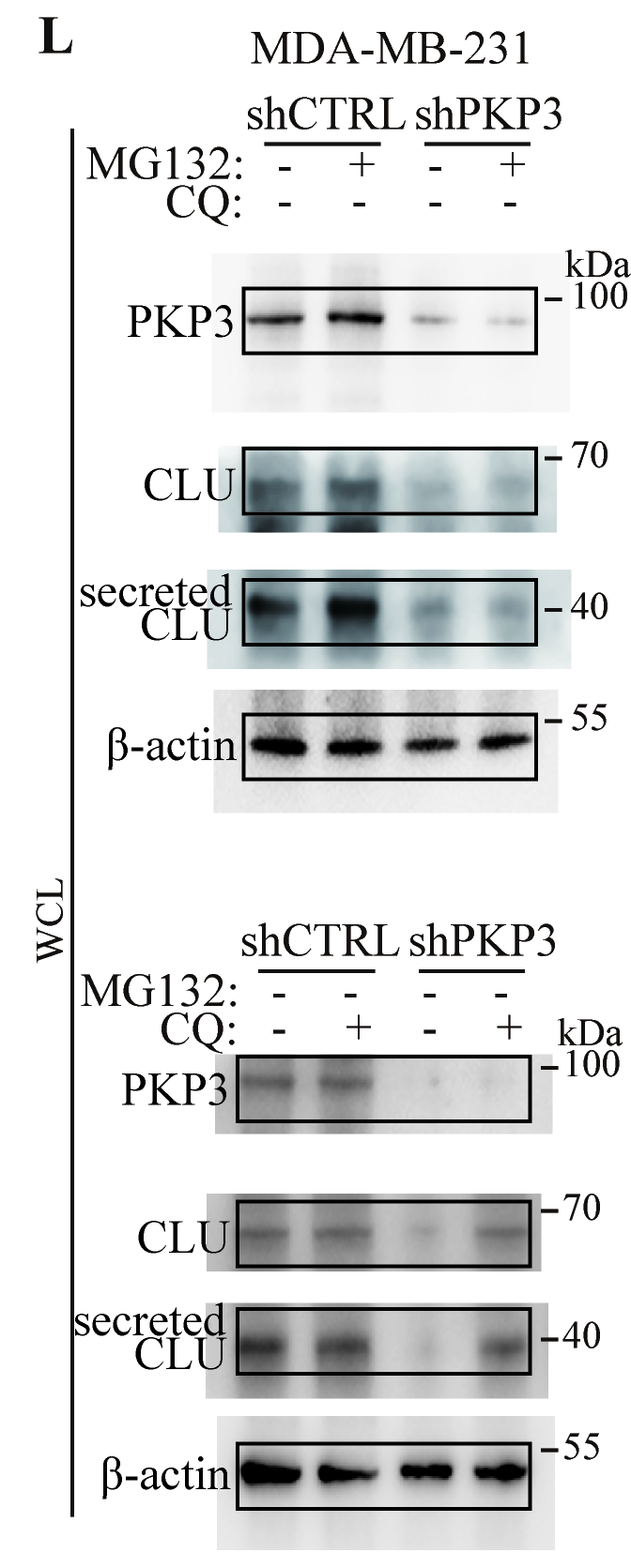

Supplement: Supplementary file 16 — Figure EV4 Source Data [file 44318_2025_661_MOESM16_ESM.zip › Figure EV4/Figure EV4L/Figure EV4L.tif]

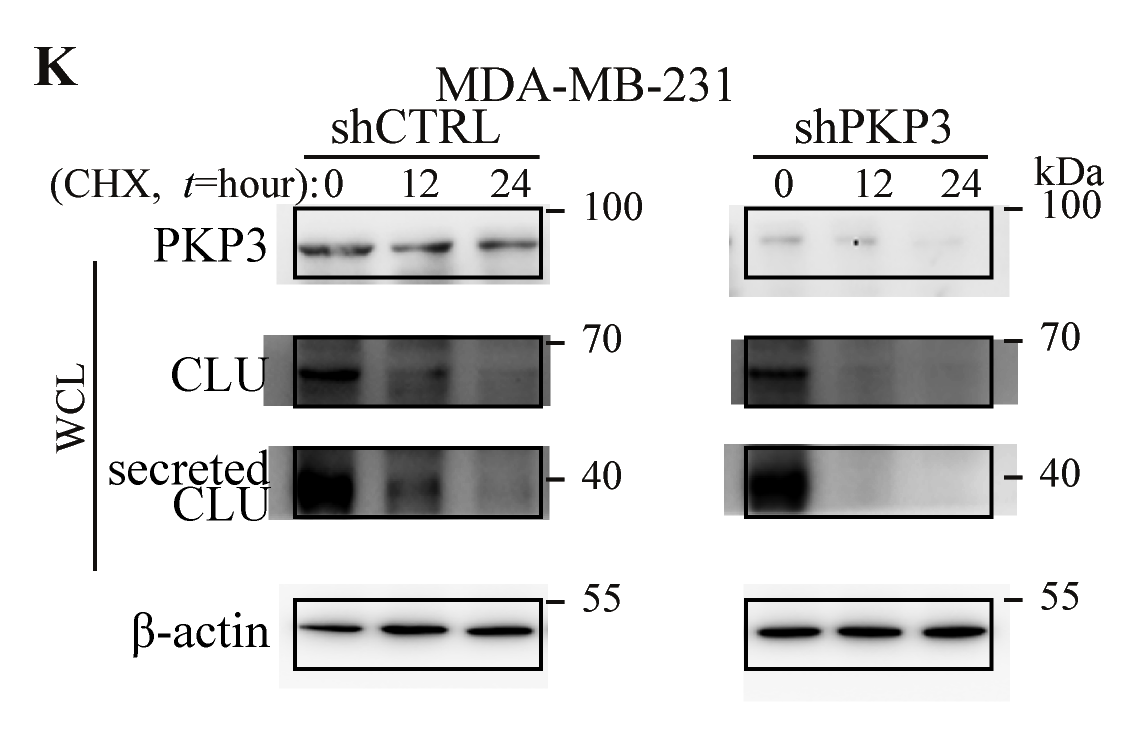

Supplement: Supplementary file 16 — Figure EV4 Source Data [file 44318_2025_661_MOESM16_ESM.zip › Figure EV4/Figure EV4K/Figure EV4K.tif]

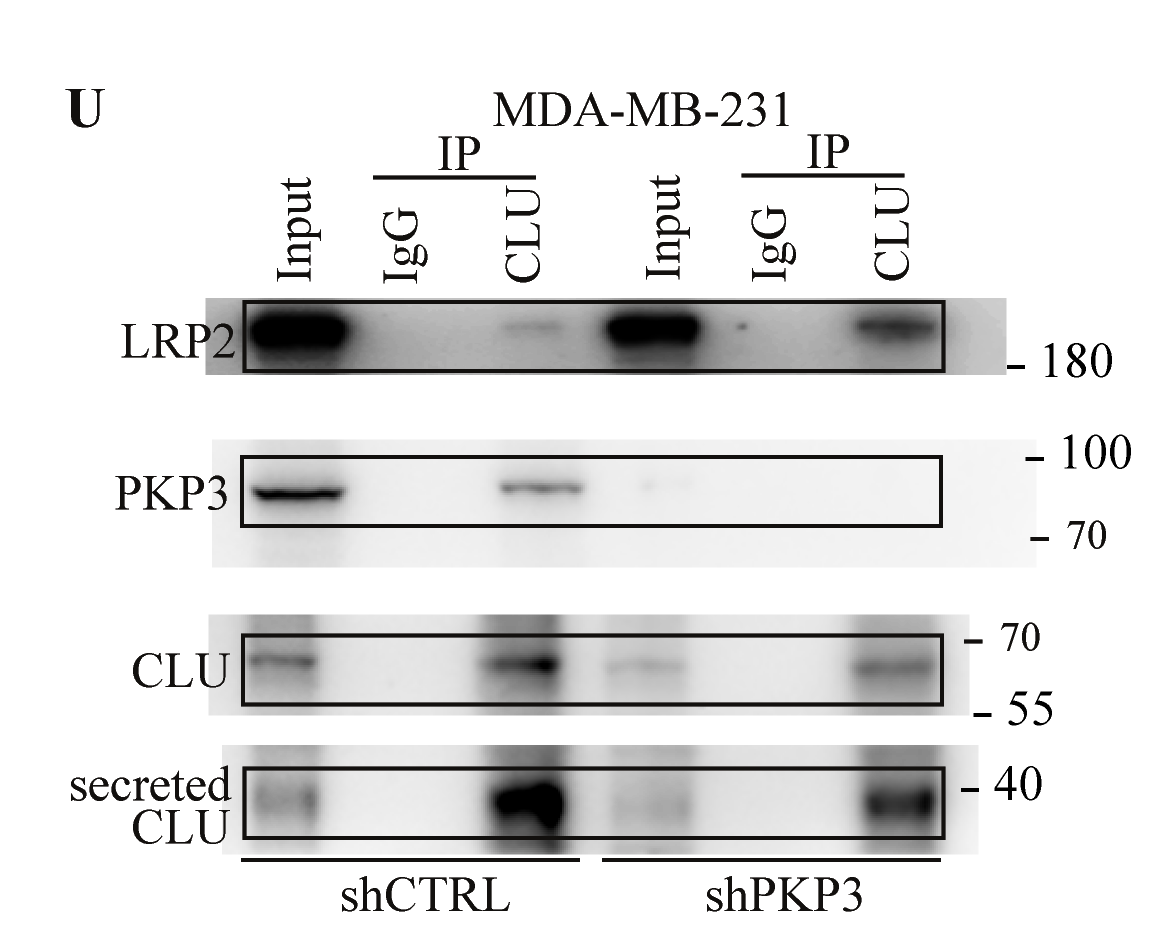

Supplement: Supplementary file 16 — Figure EV4 Source Data [file 44318_2025_661_MOESM16_ESM.zip › Figure EV4/Figure EV4U/Figure EV4U.tif]

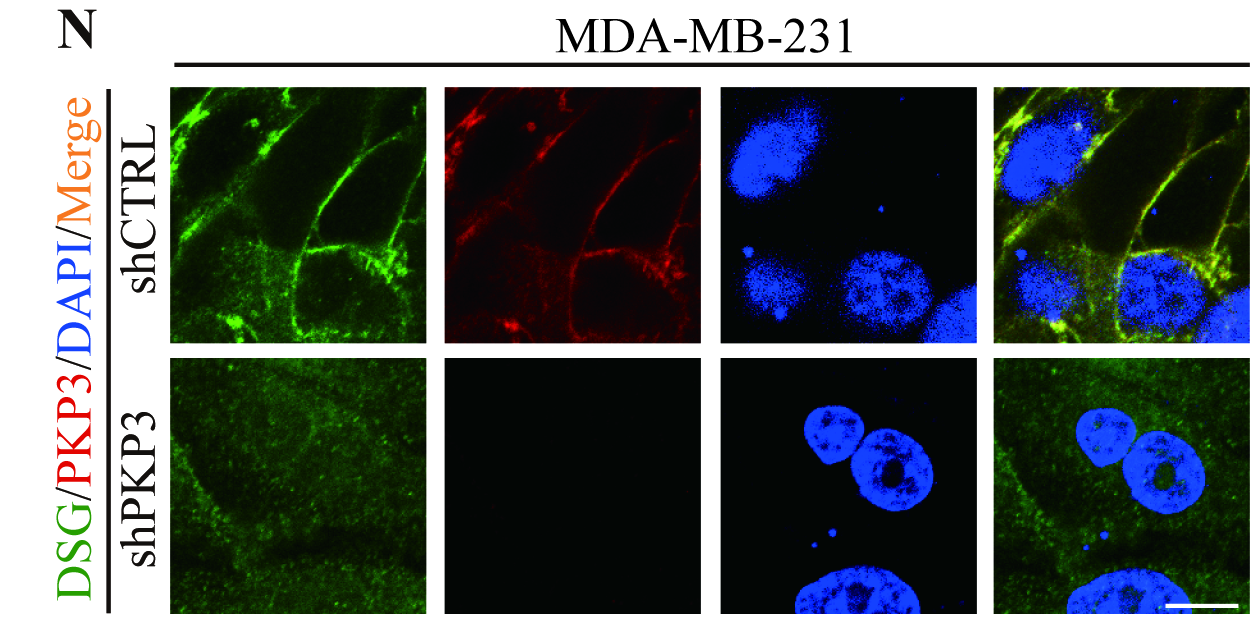

Supplement: Supplementary file 16 — Figure EV4 Source Data [file 44318_2025_661_MOESM16_ESM.zip › Figure EV4/Figure EV4N/Figure EV4N.tif]

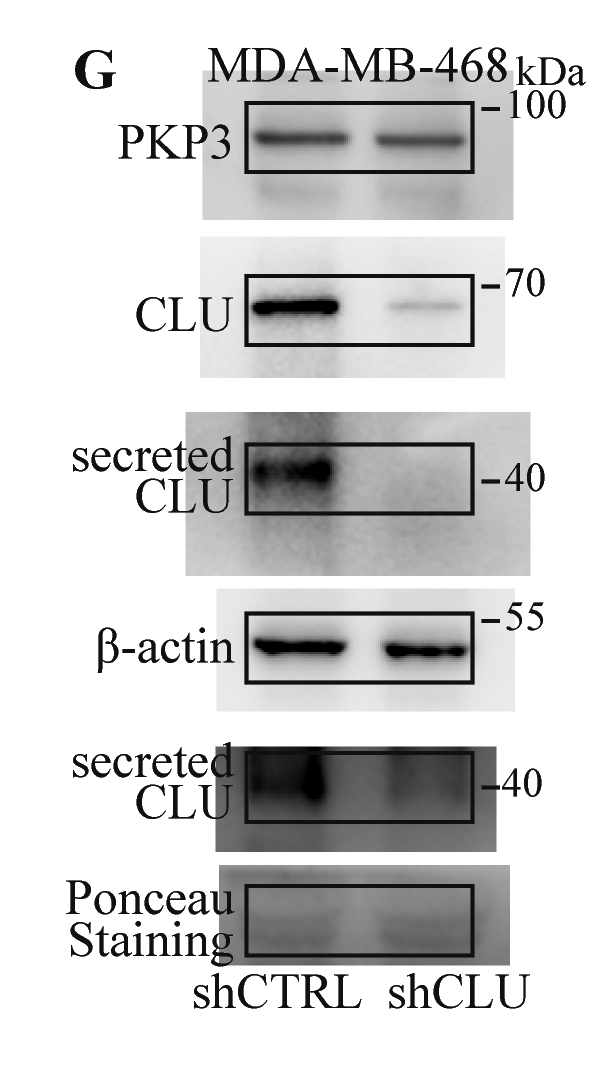

Supplement: Supplementary file 16 — Figure EV4 Source Data [file 44318_2025_661_MOESM16_ESM.zip › Figure EV4/Figure EV4G/Figure EV4G.tif]

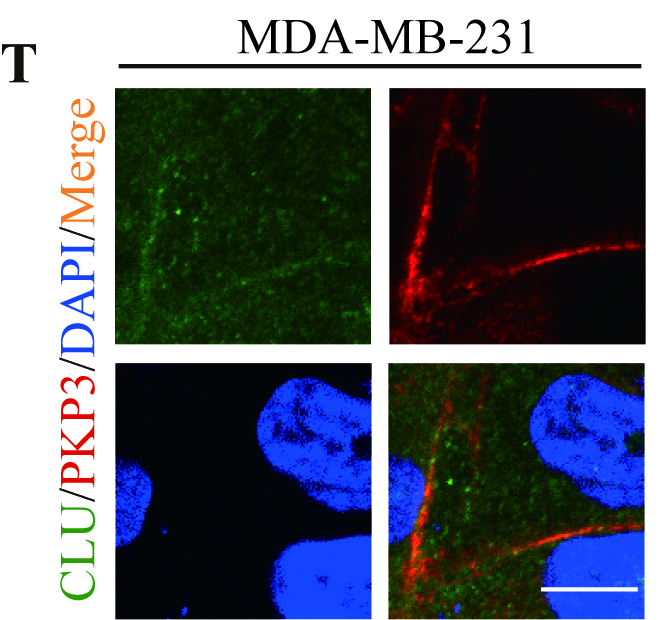

Supplement: Supplementary file 16 — Figure EV4 Source Data [file 44318_2025_661_MOESM16_ESM.zip › Figure EV4/Figure EV4T/Figure EV4T.tif]

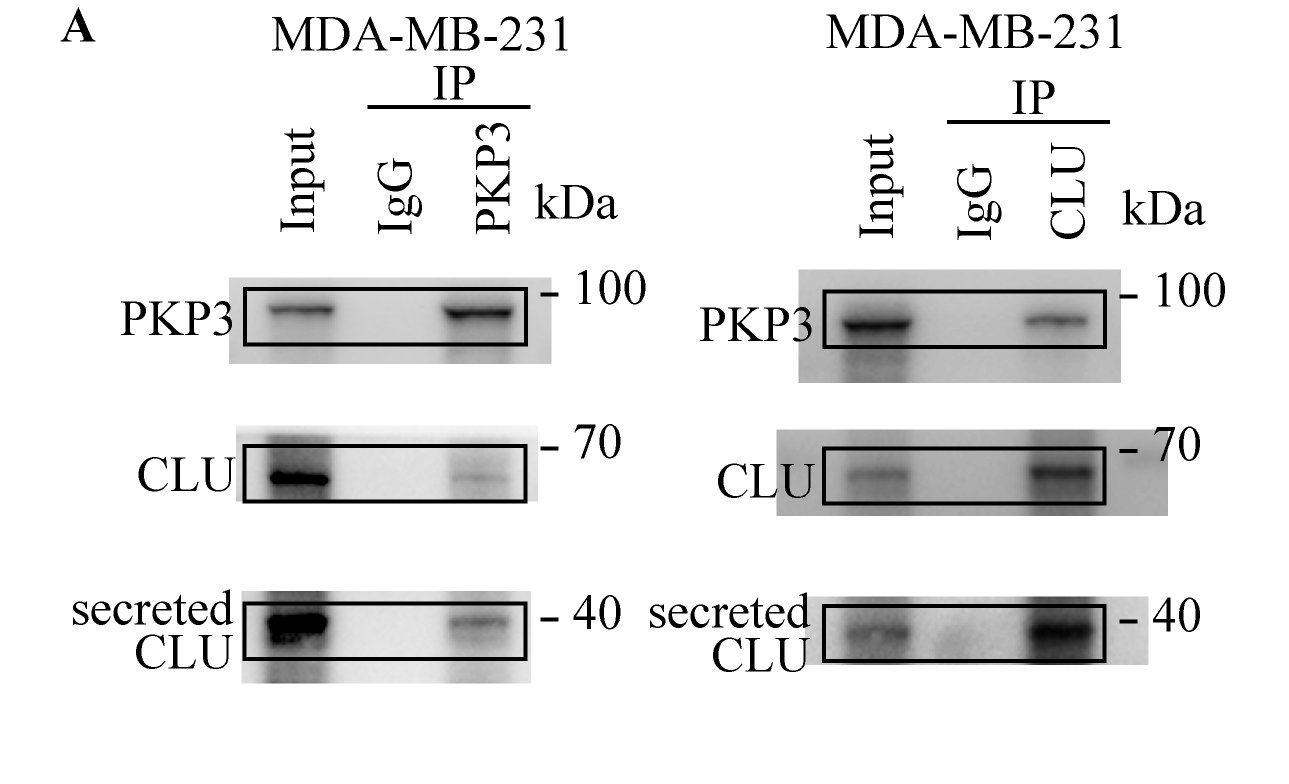

Supplement: Supplementary file 16 — Figure EV4 Source Data [file 44318_2025_661_MOESM16_ESM.zip › Figure EV4/Figure EV4A/Figure EV4A.tif]

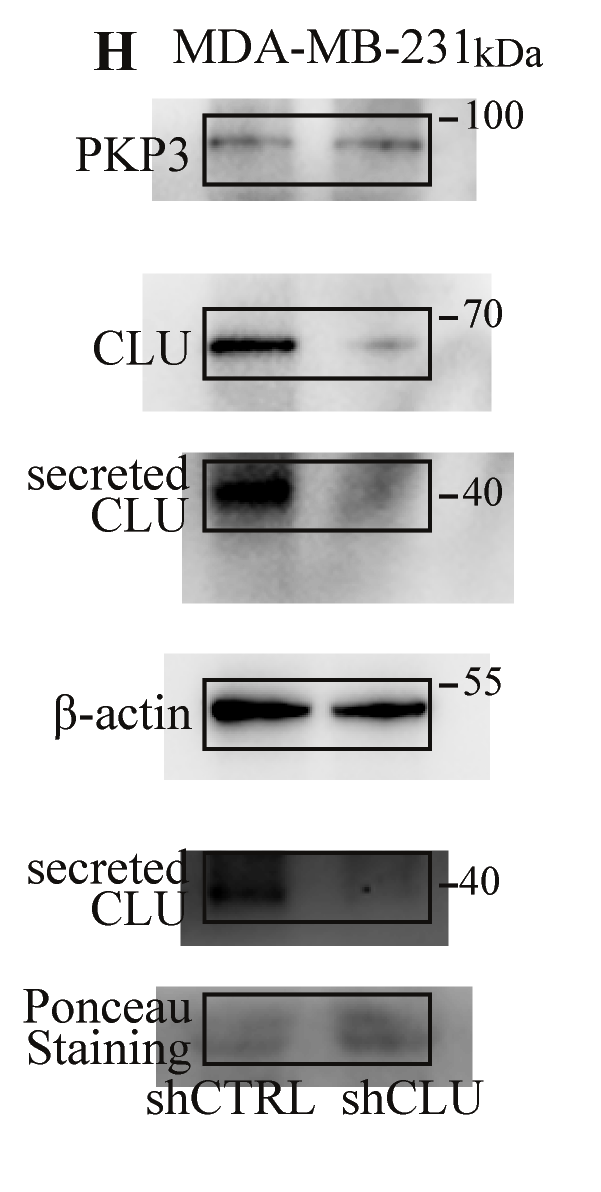

Supplement: Supplementary file 16 — Figure EV4 Source Data [file 44318_2025_661_MOESM16_ESM.zip › Figure EV4/Figure EV4H/Figure EV4H.tif]

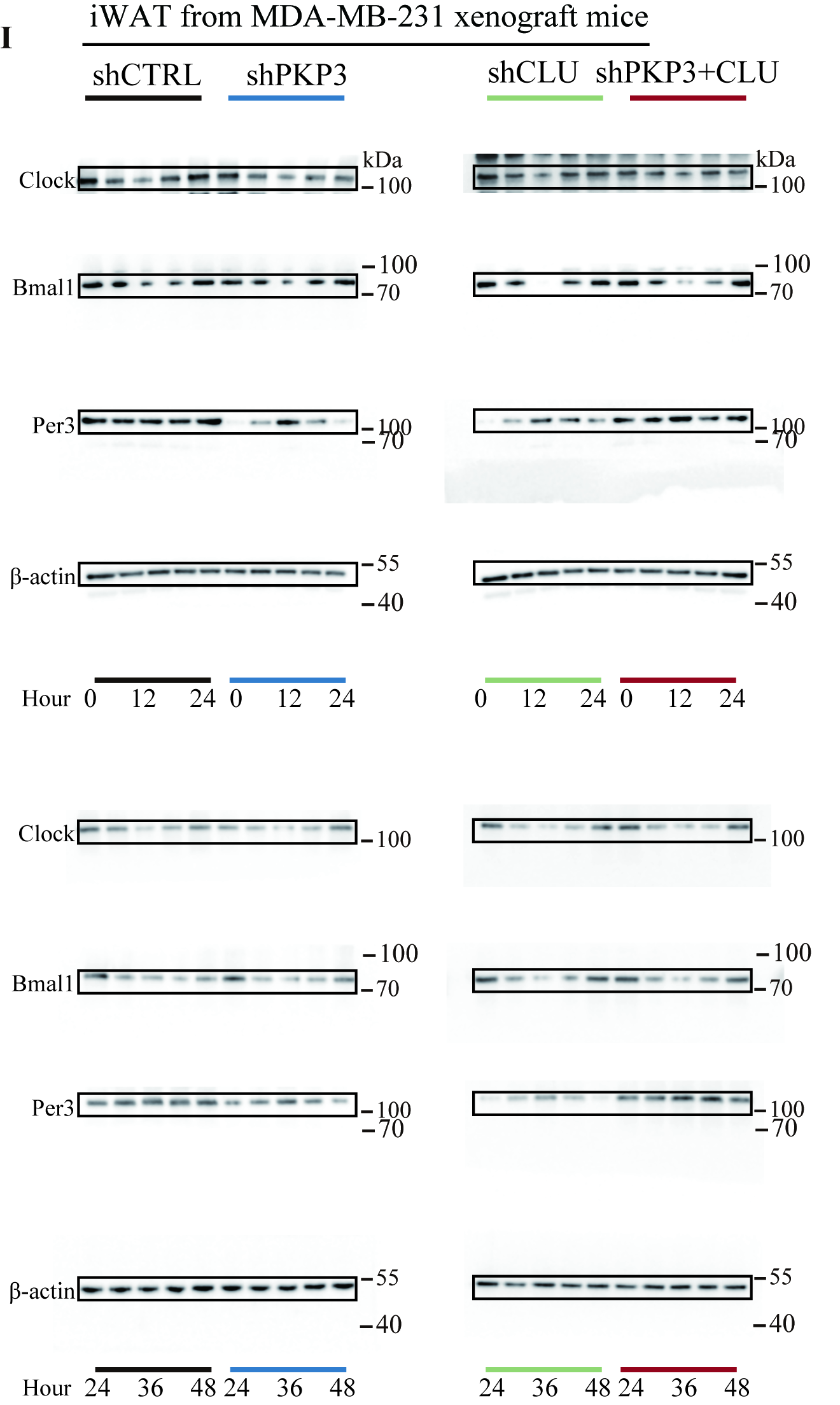

Supplement: Supplementary file 17 — Figure EV5 Source Data [file 44318_2025_661_MOESM17_ESM.zip › Figure EV5/Figure EV5I/Figure EV5I.tif]

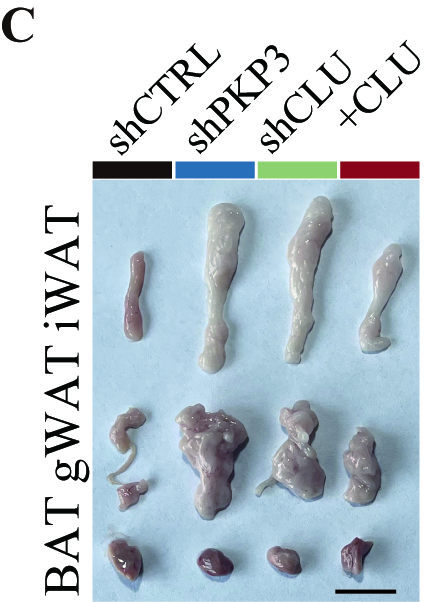

Supplement: Supplementary file 17 — Figure EV5 Source Data [file 44318_2025_661_MOESM17_ESM.zip › Figure EV5/Figure EV5C/Figure EV5C.tif]
