# Supplementary material for: Tumor-secreted clusterin promotes cachectic fat wasting via disrupting circadian gene expression and adipogenesis
Source: EMBO J. 2025 Dec 17;45(3):856–78. doi: 10.1038/s44318-025-00661-4 (PMC12864892; doi:10.1038/s44318-025-00661-4)
Supplement: Supplementary file 18 — Expanded View Figures [file 44318_2025_661_MOESM18_ESM.pdf]

Expanded View Figures

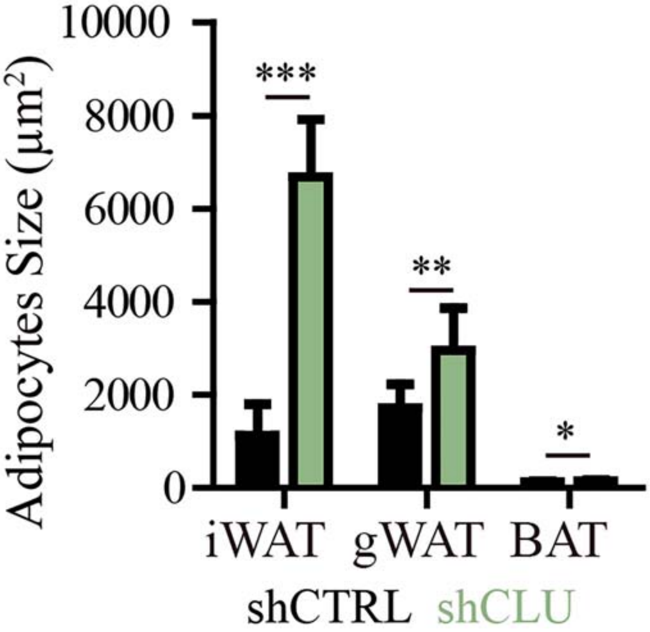

Figure EV1. Related to Fig. 1. Statistical analysis of H&E staining of iWAT, gWAT, BAT in Fig. 1G (shCTRL vs shCLU: iWAT, $P < 0.0001$; gWAT, $P = 0.0014$; BAT, $P = 0.0229$; $n = 10$ per group). The data are presented as mean ± SEM. *$p < 0.05$, **$p < 0.01$, ***$p < 0.001$. Statistical comparisons between groups were performed using unpaired two-tailed Student's t tests. Source data are available online for this figure.

 © The Author(s)

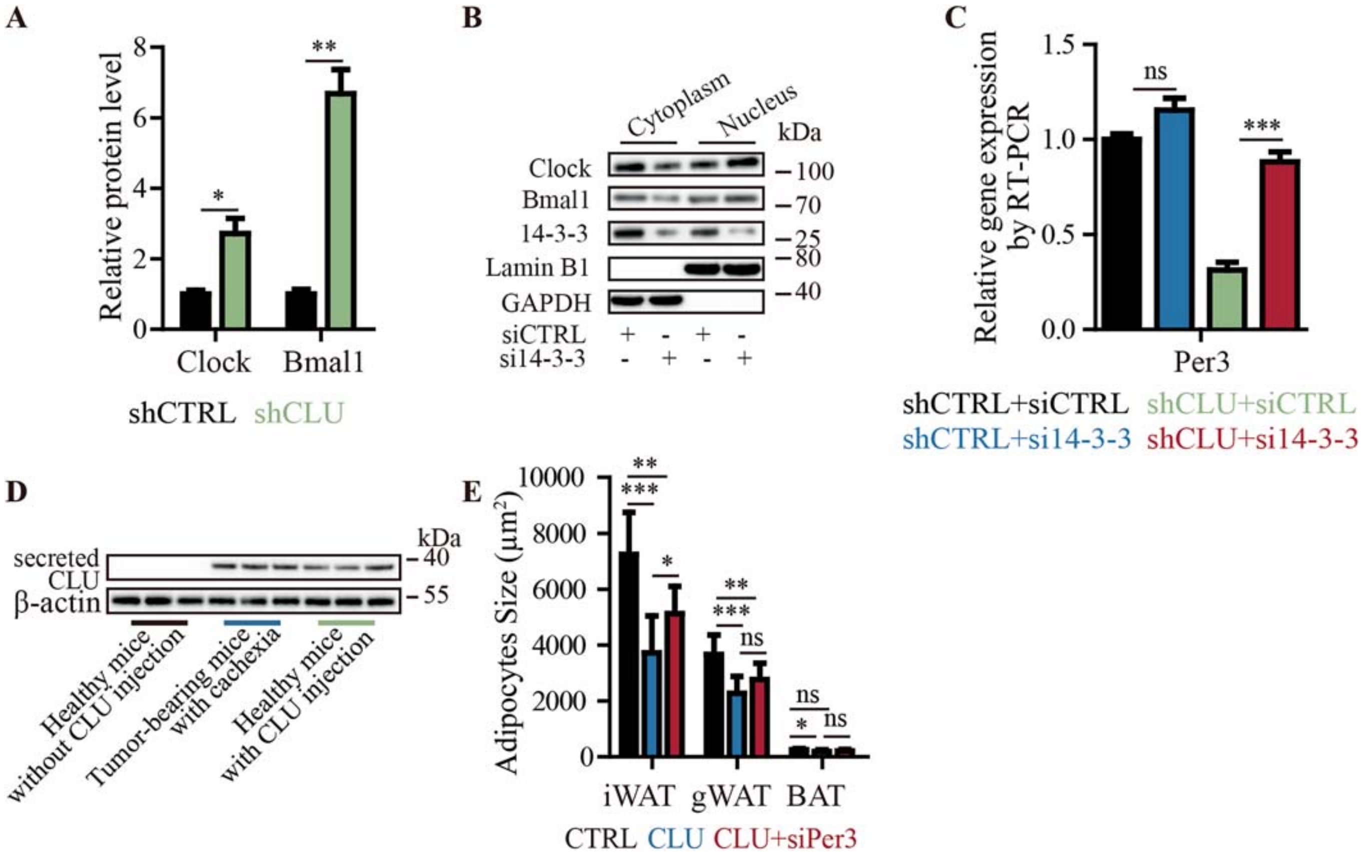

Figure EV2. **Related to** Fig. 2. (**A**) Quantification of western blot by Co-IP assays in Fig. 2F (shCTRL vs shCLU: Clock, $P = 0.0181$; Bmal1, $P = 0.0012$; $n = 3$ per group). (**B**) Representative Western blot images of a nucleocytoplasmic separation assay showing the levels of cytoplasmic and nuclear Clock, Bmal1, and 14-3-3 in iWAT cells with and without 14-3-3 knockdown in mice bearing CLU knockdown tumors. (**C**) Per3 expression in iWAT cells with and without 14-3-3 knockdown by RT-qPCR. The shCLU and shCTRL stand mice bearing tumor with and without CLU knockdown, respectively (shCTRL+siCTRL vs shCTRL+si14-3-3: *Per3*, $P = 0.0691$; shCLU+siCTRL vs shCLU+si14-3-3: *Per3*, $P = 0.0001$; $n = 4$ per group). (**D**) circulating CLU levels in iWAT of mice by western blot. (**E**) Statistical analysis of H&E staining of iWAT, gWAT, BAT in Fig. 2L (iWAT: CTRL vs CLU, $P < 0.0001$; CTRL vs CLU+siPer3, $P = 0.0016$; CLU vs CLU+siPer3, $P = 0.0151$; gWAT: CTRL vs CLU, $P = 0.0001$; CTRL vs CLU+siPer3, $P = 0.0057$; CLU vs CLU+siPer3, $P = 0.0819$; BAT: CTRL vs CLU, $P = 0.0137$; CTRL vs CLU+siPer3, $P = 0.0511$; CLU vs CLU+siPer3, $P = 0.5122$; $n = 10$ per group). The data are presented as mean ± SEM. $^*p < 0.05$, $^{**}p < 0.01$, $^{***}p < 0.001$, ns for not significant. Statistical comparisons between groups were performed using unpaired two-tailed Student's t tests. Source data are available online for this figure.

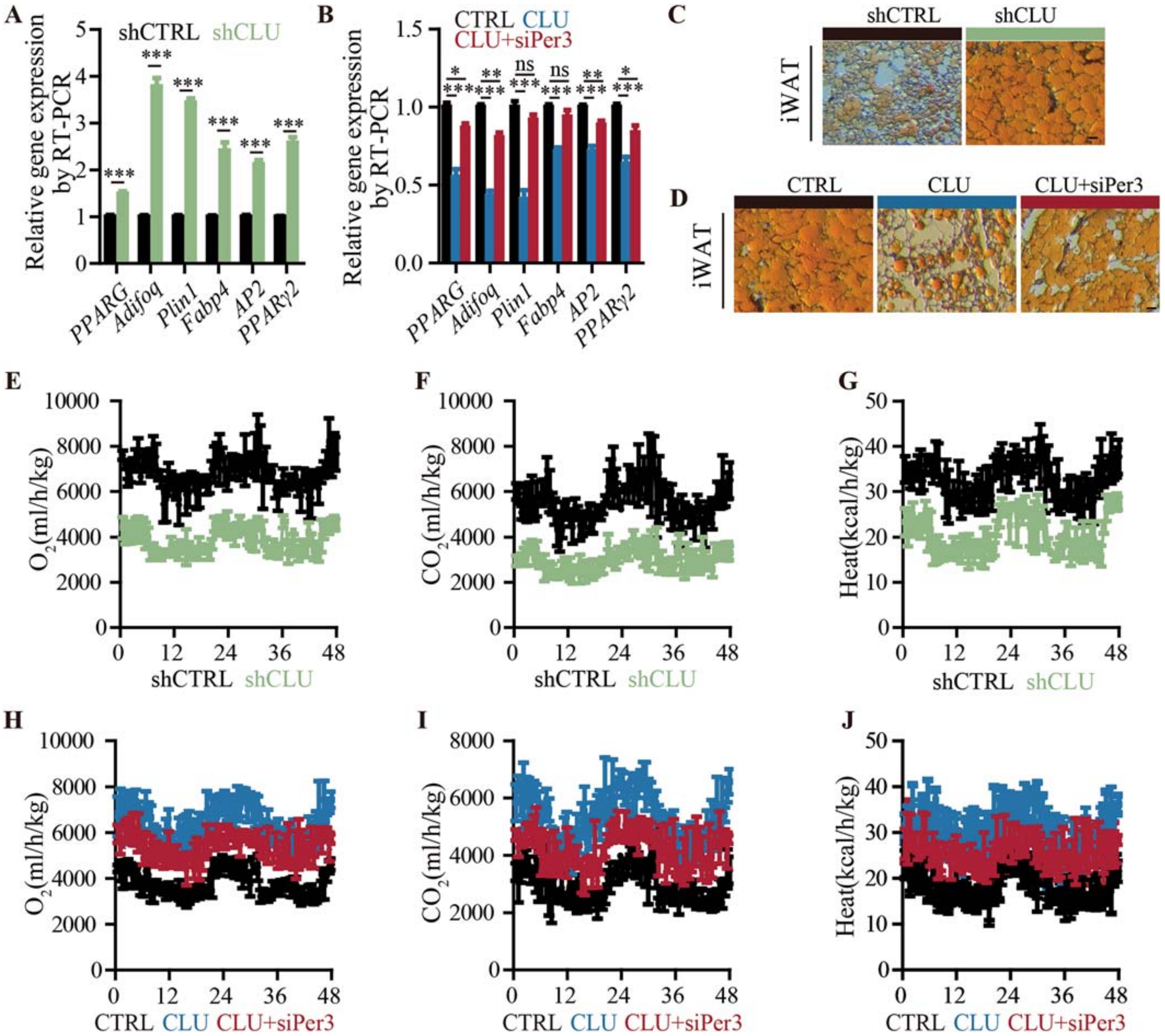

Figure EV3. The CLU-Per3 axis contribute to adipogenesis and energy expenditure.

(A) Adipogenesis-related gene expression by RT-qPCR in iWAT of MDA-MB-468 tumor-bearing mice with indicated treatment (shCTRL vs shCLU: *PPARG*, $P = 0.0003$; *Adifoq*, $P < 0.0001$; *Plin1*, $P < 0.0001$; *Fabp4*, $P = 0.0002$; *AP2*, $P < 0.0001$; *PPARγ2*, $P < 0.0001$; $n = 4$ per group). (**B**) Adipogenesis-related gene expression by RT-qPCR in iWAT of healthy nude mice with indicated treatment (CTRL vs CLU: *PPARG*, $P = 0.0002$; *Adifoq*, $P < 0.0001$; *Plin1*, $P = 0.0002$; *Fabp4*, $P = 0.0001$; *AP2*, $P = 0.0002$; *PPARγ2*, $P = 0.0003$; CTRL vs CLU+siPer3: *PPARG*, $P = 0.0142$; *Adifoq*, $P = 0.0018$; *Plin1*, $P = 0.1590$; *Fabp4*, $P = 0.2300$; *AP2*, $P = 0.0088$; *PPARγ2*, $P = 0.0163$; $n = 10$ per group). (**C**, **D**) Adipogenesis by using Oil Red O staining in iWAT of mice. (**E**, **H**) Oxygen consumption, (**F**, **I**) carbon dioxide generation and (**G**, **J**) heat production by metabolic cages in mice ($n = 3$ per group). The data are presented as mean ± SEM. *$p < 0.05$, **$p < 0.01$, ***$p < 0.001$, ns for not significant. Statistical comparisons between groups were performed using unpaired two-tailed Student's t tests. Source data are available online for this figure.

 © The Author(s)

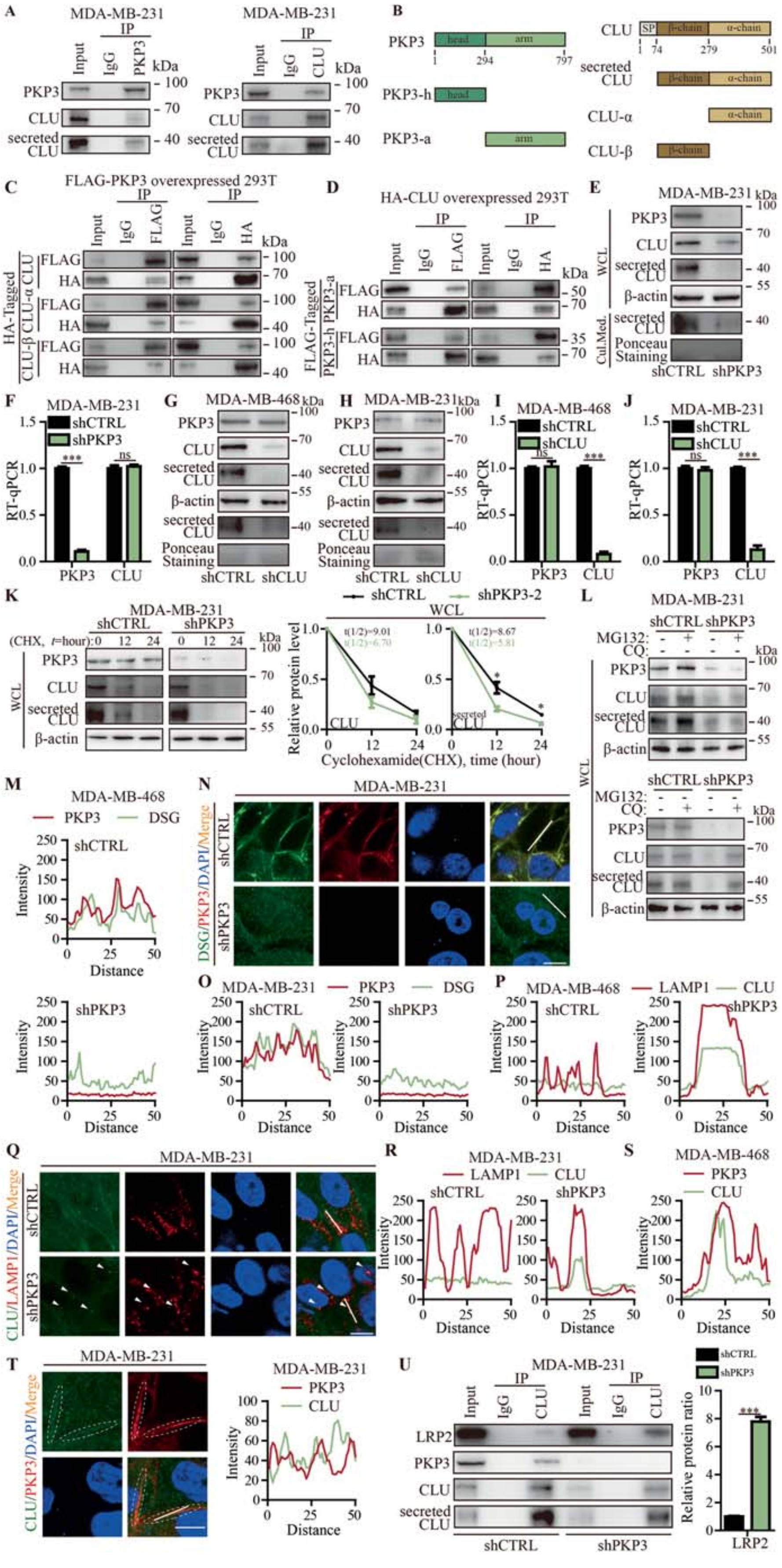
A
MDA-MB-231
IP
Input IgG PKP3 kDa
PKP3 - 100
CLU - 70
secreted CLU - 40
MDA-MB-231
IP
Input IgG CLU kDa
B
PKP3 head arm
PKP3-h head
PKP3-a arm
CLU SP β-chain α-chain
secreted CLU
CLU-α
CLU-β
C
FLAG-PKP3 overexpressed 293T
HA-Tagged
D
HA-CLU overexpressed 293T
FLAG-Tagged
E
MDA-MB-231
WCL
Cul.Med.
Ponceau Staining
shCTRL shPKP3
β-actin
F
MDA-MB-231
shCTRL
shPKP3
RT-qPCR
G
MDA-MB-468
shCTRL shCLU
H
MDA-MB-231
I
MDA-MB-468
J
MDA-MB-231
K
MDA-MB-231
(CHX, t=hour)
shPKP3-2
Relative protein level
Cyclohexamide(CHX), time (hour)
L
MG132:
CQ:
M
MDA-MB-468
DSG
Intensity
Distance
N
DSG/PKP3/DAPI/Merge
O
P
LAMP1
Q
CLU/LAMP1/DAPI/Merge
R
S
T
CLU/PKP3/DAPI/Merge
U
LRP2
Relative protein ratio

Figure EV4. CLU stabilization by PKP3 in TNBC cancer cells.

(**A**) Representative Western blot images showing PKP3, CLU and secreted CLU levels in Co-IP assays using TNBC MDA-MB-231 cells. (**B**) Schematic representation of full-length PKP3, CLU, secreted CLU and their domain truncates. (**C**) Representative Western blot images of Co-IP assays using 293 T cells with ectopic overexpression of FLAG-PKP3 and HA-CLU or its domain truncates (HA-CLU-α, HA-CLU-β). (**D**) Representative Western blot images of Co-IP assays using 293 T cells with ectopic overexpression of HA-CLU and PKP3 truncates (FLAG-PKP3-a, FLAG-PKP3-h). (**E**) Representative Western blot images showing PKP3, CLU and secreted CLU protein levels in MDA-MB-231 cells and secreted CLU protein levels in cell culture medium (Cul. Med.) upon PKP3 knockdown compared to controls. (**F**) PKP3 and CLU mRNA levels in MDA-MB-231 cells by RT-qPCR. (**G–J**) PKP3 and CLU mRNA and PKP3, CLU and secreted CLU protein levels in TNBC MDA-MB-468 and MDA-MB-231 cancer cells in cell culture medium (Cul. Med.) upon CLU knockdown compared to controls. (**G**, **H**) Representative Western blot images. (**I**, **J**) RT-qPCR ($n = 4$ per group). (**K**) Representative Western blot images and their quantification of PKP3, CLU and secreted CLU protein levels in MDA-MB-231 cells upon PKP3 knockdown compared to control after CHX treatment (shCTRL vs shPKP3-2: secreted CLU, 12-hour, $P = 0.0261$; 24-hour, $P = 0.0137$). (**L**) Representative Western blot images showing PKP3, CLU and secreted CLU protein levels in cancer cells upon MG132 or CQ treatment in PKP3 knockdown MDA-MB-231 cells compared to control. (**M**) Colocalization analysis of Fig. 3F. (**N**) Representative immunofluorescence images and (**O**) colocalization analysis showing PKP3 and DSG localization in MDA-MB-231 cells after PKP3 knockdown compared to the control. Green for DSG, red for PKP3, blue for DAPI and orange for DSG-PKP3 colocalization. Scale bar: 10 μM. (**P**) Colocalization analysis of Fig. 3G. (**Q**) Representative immunofluorescence images and (**R**) colocalization analysis showing PKP3 and CLU localization in MDA-MB-231 cells. White dashed lines highlight the intercellular plaque regions, green for CLU, red for PKP3, blue for DAPI, orange for CLU-PKP3 colocalization. Scale bar: 10 μM. (**S**) Colocalization analysis of Fig. 3H. (**T**) Representative immunofluorescence images and colocalization analysis showing CLU and LAMP1 localization in MDA-MB-231 cells upon PKP3 knockdown compared to the control. Green for CLU, red for LAMP1, blue for DAPI and orange for CLU-LAMP1 colocalization. Scale bar: 10 μM. (**U**) Representative Western blot images and quantification of LRP2, PKP3, CLU and secreted CLU levels in Co-IP assays using MDA-MB-231 cells upon PKP3 knockdown compared to the control (shCTRL vs shPKP3: *LRP2*, $P < 0.0001$; $n = 3$ per group). The data are presented as mean ± SEM (n = [X] biologically independent samples). $^*p < 0.05$, $^{***}p < 0.001$, ns for not significant. Statistical comparisons between groups were performed using unpaired two-tailed Student's t tests. Source data are available online for this figure.

 © The Author(s)

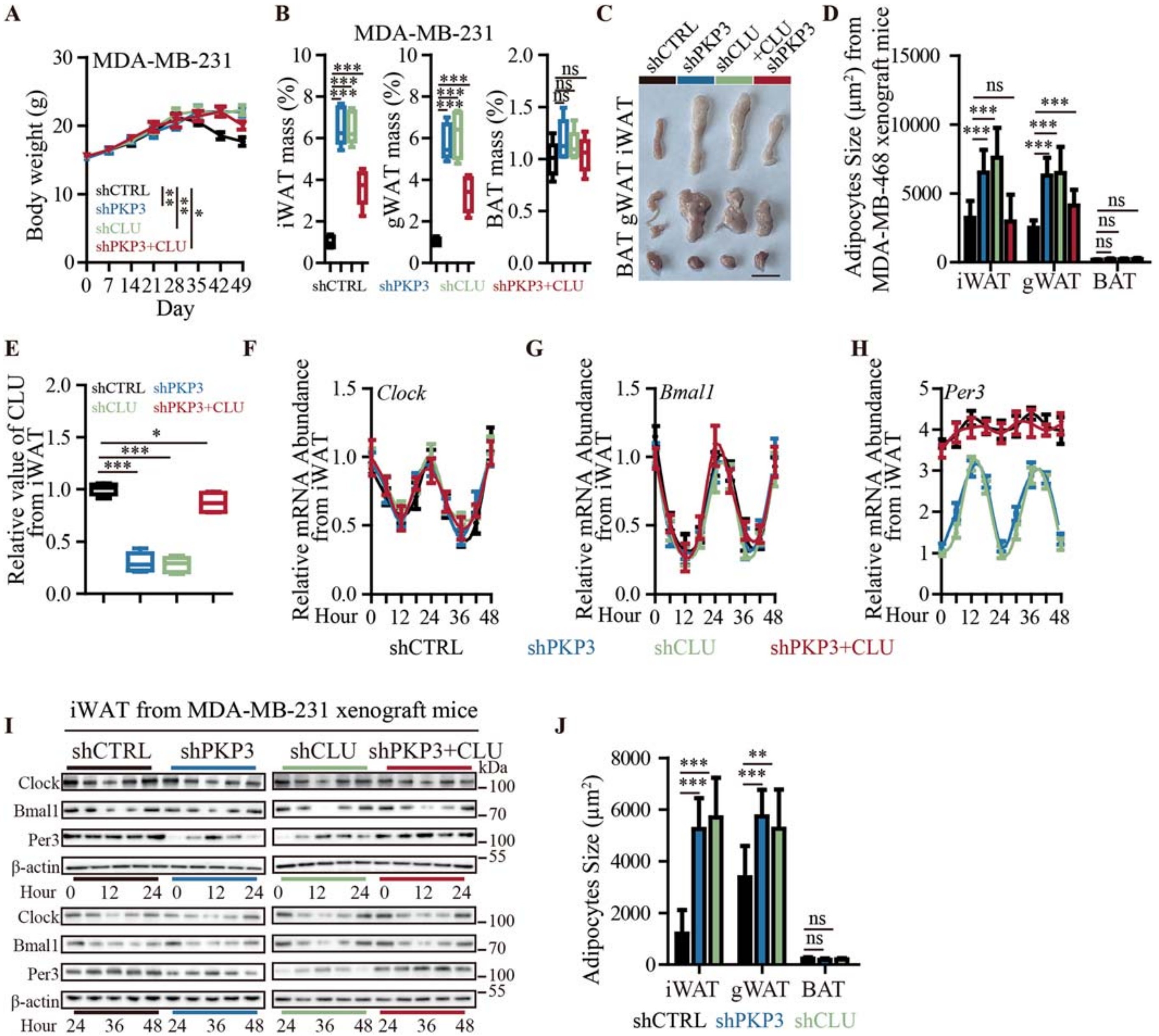

Figure EV5. Disruption of WAT's circadian rhythm by tumor PKP3-CLU axis for cachectic fat mass loss.

(**A–I**) MDA-MB-231 tumor-bearing mice with PKP3 knockdown, CLU knockdown, or PKP3 knockdown combined with ectopic overexpression of CLU ($n = 5$ per group): (**A**) Body weight (shCTRL vs shPKP3, $P = 0.0015$; shCTRL vs shCLU, $P = 0.0012$; shCTRL vs shPKP3+CLU, $P = 0.0260$; $n = 5$ per group); (**B**) Weight change of iWAT, gWAT, BAT (iWAT: shCTRL vs shPKP3, $P < 0.0001$; shCTRL vs shCLU, $P < 0.0001$; shCTRL vs shPKP3+CLU, $P = 0.0003$; gWAT: shCTRL vs shPKP3, $P < 0.0001$; shCTRL vs shCLU, $P < 0.0001$; shCTRL vs shPKP3+CLU, $P < 0.0001$; BAT: shCTRL vs shPKP3, $P = 0.1656$; shCTRL vs shCLU, $P = 0.1322$; shCTRL vs shPKP3+CLU, $P = 0.7618$; $n = 5$ per group), in a box-plot, the center line represents the median of the data, while the lower and upper limits of the box correspond to the first quartile and third quartile, respectively; (**C**) Representative iWAT, gWAT, BAT photos, Scale bar: 1 cm; (**D**) Statistical analysis of H&E staining of iWAT, gWAT, BAT in Fig. 4D (iWAT: shCTRL vs shPKP3, $P < 0.0001$; shCTRL vs shCLU, $P < 0.0001$; shCTRL vs shPKP3+CLU, $P = 0.7042$; gWAT: shCTRL vs shPKP3, $P < 0.0001$; shCTRL vs shCLU, $P < 0.0001$; shCTRL vs shPKP3+CLU, $P = 0.0009$; BAT: shCTRL vs shPKP3, $P = 0.2816$; shCTRL vs shCLU, $P = 0.1173$; shCTRL vs shPKP3+CLU, $P = 0.0980$; $n = 10$ per group). (**E**) Tumor-secreted CLU levels in iWAT by ELISA assay (shCTRL vs shPKP3, $P < 0.0001$; shCTRL vs shCLU, $P < 0.0001$; shCTRL vs shPKP3+CLU, $P = 0.0277$; $n = 5$ per group); (**F–I**) CLOCK, BMAL1, PER3 expression in WAT in a 48-hour cycle by (F-H) RT-qPCR ($n = 4$ per group) and (*I*) Western blot assay. (**J**) Statistical analysis of H&E staining of iWAT, gWAT, BAT in Fig. 6G (iWAT: shCTRL vs shPKP3, $P < 0.0001$; shCTRL vs shCLU, $P < 0.0001$; gWAT: shCTRL vs shPKP3, $P = 0.0002$; shCTRL vs shCLU, $P = 0.0068$; BAT: shCTRL vs shPKP3, $P = 0.0745$; shCTRL vs shCLU, $P = 0.1963$; $n = 3$ per group). The data are presented as mean ± SEM ($n =$ [X] biologically independent samples). *$p < 0.05$, **$p < 0.01$, ***$p < 0.001$, ns for not significant. Statistical comparisons between groups were performed using unpaired two-tailed Student's t tests. Source data are available online for this figure.

© The Author(s)
